# Bridging knowledge and practice gaps in antimicrobial stewardship among veterinary professionals in Zambia’s poultry sector: a mixed-methods pre–post intervention study

**DOI:** 10.3389/frabi.2026.1898867

**Published:** 2026-07-30

**Authors:** Chikwanda Chileshe, Chisoni Mumba, Ricky Chazya, William Ngosa, Steward Mudenda, Victor Chishimba, Taona Sinyawa, Kenneth Chawinga, Misheck Shawa, Geoffrey Mainda, Iyamvwa Muchepa, Joseph Yamweka Chizimu, Gilbert Nchima, Bernard Mudenda Hang’ombe, Fusya Goma, Walter Muleya, Ntombi B. Mudenda

**Affiliations:** 1 School of Veterinary Medicine, University of Zambia, Lusaka, Zambia; 2 Zambia National Public Health Institute, Antimicrobial Resistance Coodinating Committee, Lusaka, Zambia; 3 Central Veterinary Research Institute, Ministry of Fisheries and Livestock, Lusaka, Zambia; 4 Department of Veterinary Services, Ministry of Fisheries and Livestock, Lusaka, Zambia; 5 Hokudai Centre for Zoonosis Control in Zambia, Hokkaido University, Lusaka, Zambia; 6 Food and Agriculture Organization of the United Nations (FAO), Lusaka, Zambia; 7 Directorate of Research and Innovation, Office of the Vice-Chancellor for Research and Innovation, Copperbelt University, Kitwe, Zambia

**Keywords:** antibiotic resistance, antimicrobial stewardship, attitude, knowledge, one health, practices, veterinarians, veterinary paraprofessionals

## Abstract

Antimicrobial resistance (AMR) poses a major threat to animal health, food security, and public health globally, with poultry production systems recognized as important contributors to antimicrobial use. Veterinary professionals and veterinary paraprofessionals play a central role in antimicrobial stewardship through prescribing practices, farmer education and disease prevention strategies. This mixed-methods pre–post intervention study assessed changes in knowledge, attitudes and practices related to antimicrobial stewardship among veterinary personnel involved in poultry production in selected districts of Zambia, before and after implementation of antimicrobial stewardship interventions under the Combating antimicrobial resistance and antimicrobial residues in Zambian Poultry (CAZAAP) project. Baseline and endline quantitative KAP surveys were conducted among veterinary professionals and paraprofessionals in Lusaka Province (Chongwe, Chilanga district) and Copperbelt Province (Ndola, Kitwe districts), complemented by qualitative key informant interviews exploring contextual drivers of antimicrobial use and stewardship practices. Knowledge of antibiotics and AMR was generally high across both provinces, with improvements observed in correct definitions of AMR, antibiotic residues, and withdrawal periods between baseline and endline. Positive attitudes toward antimicrobial stewardship were widely reported, including recognition that AMR was an important animal and public health issue and that biosecurity and vaccination can result in reduced antibiotic use. Changes were observed in several stewardship-related practices, including reductions in inappropriate antibiotic use for viral infections, decreased prophylactic antimicrobial use, and improved biosecurity practices. However, important gaps persisted, particularly limited access to treatment guidelines, inconsistent prescription practices, and low utilization of laboratory diagnostics. Qualitative findings revealed widespread farmer self-medication, over-the-counter access to antibiotics through agrovet shops and pharmacies, weak regulatory enforcement, and economic pressures influencing antimicrobial use practices. Overall, the findings suggest that although awareness of AMR among veterinary professionals is generally high, antimicrobial stewardship challenges in Zambia’s poultry sector are driven not only by knowledge limitations but also by broader structural and governance constraints. Strengthening antimicrobial stewardship will therefore require integrated One Health interventions combining professional training, improved diagnostics, regulatory strengthening, and farmer-centered education.

## Introduction

Antimicrobial resistance is a multifaceted public health concern affecting all three sectors: the human, environment and animal sector (McEwen and Collignon, 2018; Velazquez-Meza et al., 2022; Nowbuth et al., 2023). Bacteria have the potential to develop resistance against antibiotics, which will eventually lead to the lack of effective antibiotics to treat even common infections (Nikaido, 2009; Nhung et al., 2017; Peterson and Kaur, 2018). AMR development is inextricably tied to all forms of antimicrobial use (AMU), particularly when use is sub-optimal, excessive or widespread. Avoidable practices that are considered as key drivers of AMR are AMU in animal production for growth promotion, prophylaxis, and metaphylaxis (Apata, 2009; Simoneit et al., 2015). Additionally, AMU without veterinary professional oversight is also an important driver (Kiambi et al., 2021; Mudenda et al., 2023). Therefore, the prudent use of antibiotics in animals is essential to prevent resistance development.

According to the 2024 annual registrations conducted by the Veterinary Council, Zambia had a total of 407 Veterinary Surgeons and 1, 088 Veterinary Paraprofessionals (VPPs). A significant proportion of these veterinarians and veterinary paraprofessionals are based in urban areas, especially Lusaka and the Copperbelt, while rural areas often depend more on veterinary para professionals. The Department of Veterinary Services is responsible for animal health and is headed by a director stationed at Central level. At subnational levels, the Provincial Veterinary Officer (PVO) is in charge of a province which comprises a number of districts with each district headed by a District Veterinary Officer (DVO), who is a Veterinary Surgeon by qualification. The districts comprise of small operational areas called Veterinary Camps which are headed by VPPs, holding a certificate or diploma in Animal Health. A VPP, therefore, reports to the DVO, who then reports to the PVO. who in turn reports to the Director of Veterinary Services. In addition, private practitioners contribute to veterinary service delivery but are mainly concentrated in urban areas. The VPPs are the first line staff in the provision of veterinary services to the livestock farmers in the country. Unfortunately, the ratio of veterinarians and para-veterinarians to livestock farmers in Zambia is considerable low, which poses significant challenges for veterinary service delivery, especially in remote districts (Caudell et al., 2020). A low number of veterinarians and VPPs serving livestock farmers can contribute to several challenges that could lead to AMR, such as delayed or inaccurate diagnosis, overreliance on antimicrobials, reduced access to vaccines and other alternatives, as well as lack of antimicrobial stewardship among others.

In May 2015 World Health Assembly endorsed a global action plan on antimicrobial resistance with one of the objectives being to improve awareness and understanding of antimicrobial resistance through effective communication, education and training (WHO Library Cataloguing-in-Publication Data Global Action Plan on Antimicrobial Resistance, 2015). Following this development, in 2017, Zambia developed its own multisectoral National Action Plan on AMR identifying awareness as an important objective (Multisectoral National Action Plan on Antimicrobial Resistance, 2017; Ministry of Fisheries and Livestock Main Report Republic of Zambia Ministry of Fisheries and Livestock Republic of Zambia Ministry of National Development Planning, 2018). The aims of AMR-related KAP studies for veterinarians and VPPs are to reveal misconceptions about antimicrobial use, detect inappropriate prescribing habits, and to generally assess levels of awareness about AMR and its public health implications. By assessing real-world behavior and decision-making processes of veterinary personnel, KAP studies guide the design of evidence-based interventions like: targeted educational programs, stewardship strategies aimed at promoting responsible antimicrobial use in animals. They also reveal systemic issues such as poor access to diagnostics, regulatory weaknesses by authorities, or economic pressures that influence veterinary practices.

The livestock sector, particularly intensive poultry production systems, has been identified as a major contributor to antimicrobial consumption globally. Antimicrobials are frequently used in poultry production for therapeutic, prophylactic, and metaphylactic purposes, and in some settings continue to be used for growth promotion despite increasing global restrictions (Apata, 2009; Simoneit et al., 2015). Such practices create strong selective pressure for the emergence of resistant bacteria that may spread between animals, humans, and the environment through direct contact, food chains, and environmental contamination (Kiambi et al., 2021; Mudenda et al., 2023).

In low- and middle-income countries (LMICs), antimicrobial stewardship within livestock systems is complicated by several structural challenges, including weak regulatory systems, inadequate veterinary coverage, limited diagnostic capacity, unrestricted access to veterinary drugs, and poor farmer awareness. In many African countries, antibiotics are commonly obtained without prescriptions through informal veterinary drug markets and agrovet shops, contributing to inappropriate antimicrobial use practices (Caudell et al., 2020). These conditions create important barriers to effective antimicrobial stewardship implementation.

Veterinary professionals and veterinary paraprofessionals occupy a critical position within antimicrobial stewardship systems because they influence antimicrobial prescribing practices, disease diagnosis, farmer education, vaccination programs, and biosecurity implementation. Their knowledge, attitudes, and practices therefore play a major role in determining antimicrobial use patterns within livestock production systems. However, studies conducted across sub-Saharan Africa have demonstrated that although awareness of AMR may be increasing, inappropriate prescribing behaviors and weak stewardship implementation remain common (Caudell et al., 2020; Mudenda et al., 2022; Chilawa et al., 2023).

In Zambia, poultry production represents one of the fastest-growing livestock subsectors and contributes significantly to national food security, employment, and household income. Concurrently, increasing antimicrobial use within commercial and small-scale poultry production systems has raised concerns regarding antimicrobial resistance emergence and transmission. Zambia adopted a multisectoral National Action Plan on Antimicrobial Resistance in 2017, emphasizing awareness creation, surveillance, stewardship, and strengthened regulation (WHO Library Cataloguing-in-Publication Data Global Action Plan on Antimicrobial Resistance, 2015; Multisectoral National Action Plan on Antimicrobial Resistance, 2017).

Although several KAP studies have examined antimicrobial use among livestock farmers in Zambia (Mudenda et al., 2022; Chilawa et al., 2023), relatively few studies have focused specifically on veterinary professionals and paraprofessionals, particularly using mixed-methods approaches capable of exploring both behavioral and systemic drivers of antimicrobial use. Furthermore, evidence remains limited regarding how stewardship-oriented interventions influence changes in professional knowledge, attitudes, and practices over time.

The CAZAAP project was established in 2022 in response to the growing threat of AMR associated with poultry production systems in Zambia. The project aimed to reduce antimicrobial use and antimicrobial residues by at least 30% in intensively managed broiler production systems over a three-year period through the optimisation of antimicrobial prescribing and use among veterinarians, veterinary paraprofessionals, and poultry farmers in selected districts of Lusaka and Copperbelt provinces. One of the key objectives of the project was to determine baseline and post-intervention KAPs related to antimicrobial use and stewardship among broiler farmers and veterinary personnel, in order to generate evidence to inform targeted interventions. Building on the findings of the baseline assessment, a package of targeted interventions was implemented aimed at strengthening antimicrobial stewardship and promoting the rational use of antimicrobials within the poultry sector. Key among these interventions was the development and implementation of poultry Standard Treatment Guidelines (STGs) to guide evidence-based and responsible antimicrobial use. Further interventions included Antimicrobial Stewardship (AMS) training workshops conducted for both veterinary personnel, focusing on prudent antimicrobial prescribing, AMR awareness, and compliance with national guidelines. Collectively, these interventions sought to reduce inappropriate antimicrobial use and strengthen AMR mitigation efforts at the community and service delivery levels.

This study therefore assessed baseline and endline knowledge, attitudes, and practices related to antimicrobial stewardship among veterinary professionals and veterinary paraprofessionals involved in poultry production in selected districts of Zambia. The study additionally explored broader structural, behavioral, and governance-related factors influencing antimicrobial stewardship using qualitative key informant interviews. The integrated analysis presented in this paper adopts a convergent mixed-methods approach, where quantitative and qualitative findings are brought into dialogue to achieve deeper explanatory insight than either source could provide independently. This approach aims to generate comprehensive evidence to inform future policy, capacity-building initiatives, and AMR mitigation strategies in the veterinary sector.

## Materials and methods

### Study design and intervention framework

This study employed a mixed-methods pre–post intervention design involving baseline and endline quantitative KAP surveys complemented with qualitative key informant interviews (KIIs). The study evaluated changes in antimicrobial stewardship knowledge, attitudes, and practices among veterinary personnel before and after implementation of antimicrobial stewardship interventions.

The baseline quantitative survey was conducted between March and April 2024 prior to implementation of stewardship interventions, while the endline (post-intervention) survey was conducted in December 2025. The interventions comprised three components delivered between June 2024 and November 2025. First, antimicrobial stewardship training workshops, covering prudent antimicrobial prescribing, AMR awareness, and compliance with national guidelines, were held in August 2025 and were attended by all veterinary professionals from both the Copperbelt and Lusaka study districts. Second, all veterinary paraprofessionals who participated in the KAP survey served as trainers in the farmer field schools and therefore underwent AMR and AMS training prior to delivering instruction to farmers. Third, poultry Standard Treatment Guidelines were launched and disseminated by the CAZAAP project in November 2025, with all KAP participants present at the dissemination event. The antimicrobial stewardship training workshops were delivered over one week and were facilitated by trained members of the CAZAAP project team. Exposure within the surveyed cohort was effectively complete: all responding veterinary professionals attended the workshops and all KAP participants were present at the Standard Treatment Guidelines dissemination, so the analysed sample corresponds to intervention recipients rather than a mixture of exposed and unexposed respondents.

The study reporting was guided by the Strengthening the Reporting of Observational Studies in Epidemiology (STROBE) guidelines for quantitative components and the Consolidated Criteria for Reporting Qualitative Research (COREQ) checklist for qualitative components as can be seen in **Supplementary Table 1**.

### Description of the study area

**Figure 1:**
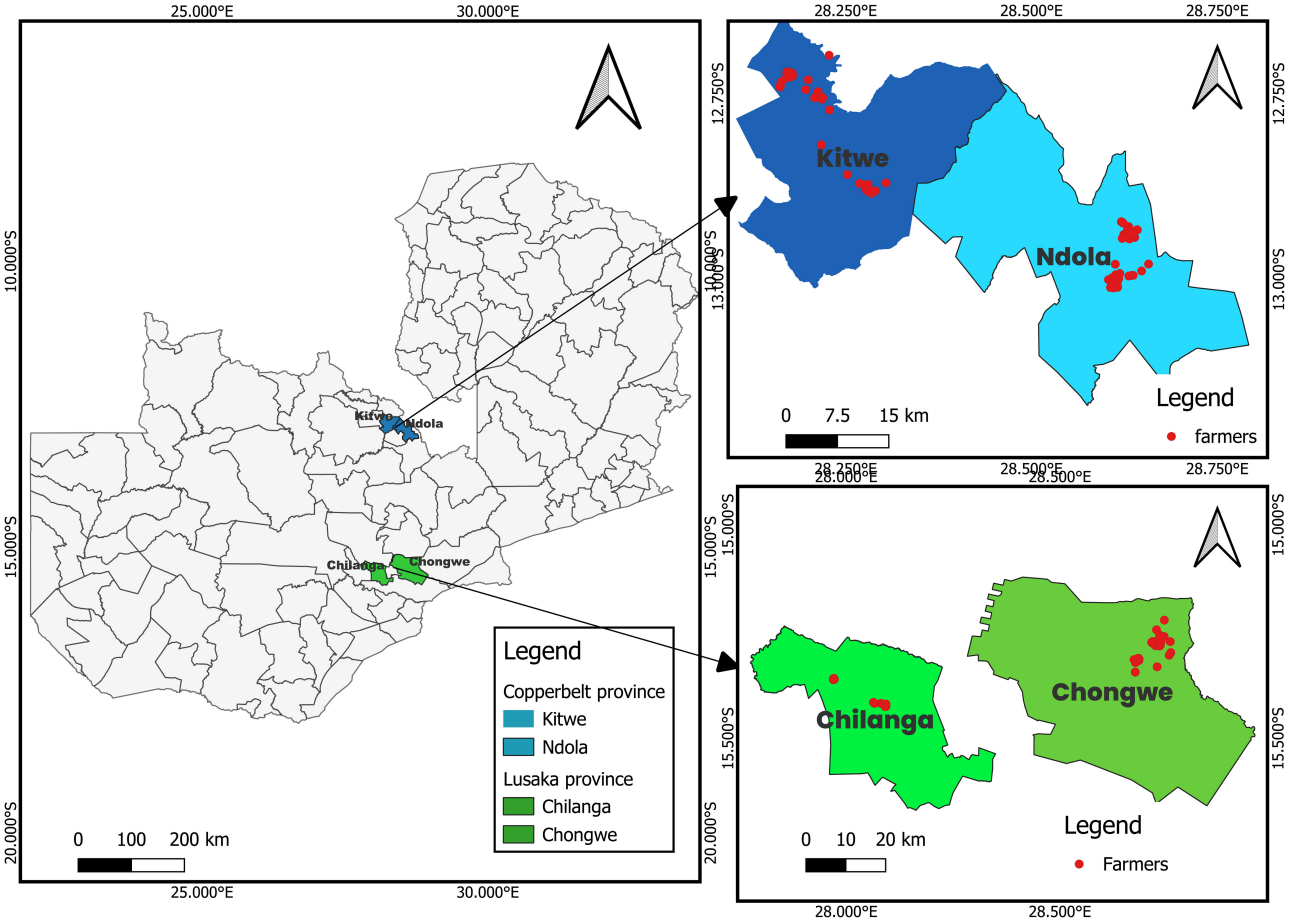
Map of Zambia showing the geographic distribution of study sites included in the KAP survey, highlighting the provinces and districts where data were collected. Developed by authors.

The project was implemented in four districts namely Chongwe and Chilanga in Lusaka Province and Ndola and Kitwe in the Copperbelt Province (**Figure 1**). These districts were selected based on their high production and consumption of poultry and poultry products as reported by the 2017/2018 Livestock and Aquaculture Census (Zambia Statistics Agency, 2022).

### Study population and sampling

The study population comprised veterinarians and VPPs actively involved in poultry health service provision within the selected districts.

Veterinary professionals were defined as individuals holding a Bachelor of Veterinary Medicine or equivalent qualification and registered with the Veterinary Council of Zambia. Veterinary paraprofessionals included veterinary assistants, animal health technicians, and animal science personnel involved in animal health service delivery in both public and private sectors.

Given the relatively small population of veterinary personnel actively involved in poultry-related veterinary services within the selected districts, exhaustive sampling was employed. All eligible veterinary personnel identified within the project implementation districts were invited to participate.

Because the study employed exhaustive sampling of all eligible veterinary personnel within the four implementation districts, the sample size was determined by the size of the available population rather than by an *a priori* power calculation; such a calculation was therefore not applicable. As a consequence, the study was adequately powered only to detect large effects, and province-level subgroup comparisons (particularly for Lusaka, n=16) are underpowered and were interpreted as exploratory.

For qualitative data collection, purposive sampling was used to identify key informants with extensive experience in veterinary practice, antimicrobial use, poultry health management, and antimicrobial stewardship as well as familiarity with the district they served in. Key informants were senior veterinary professionals, whose roles and provinces are described in the Qualitative findings. All interviews were conducted by a single trained interviewer (C.C.), a public health researcher holding a Master of Science in Public Health and a member of the CAZAAP project research team, who had completed training in qualitative interviewing techniques prior to data collection. Interviews followed a semi-structured interview guide, were conducted in person, and each interview lasted approximately 20–30 minutes. Interviews continued until no substantially new major themes emerged across informants; given the small number of senior key informants available within the project districts, the interviews are presented as a focused explanatory strand complementing the quantitative survey rather than as a claim of full thematic saturation.

### Questionnaire design and data collection

A semi-structured questionnaire was developed, consisting of four sections: the first captured demographic information; the second assessed knowledge on antibiotics and antimicrobial resistance, including sources of information and awareness levels; the third examined attitudes toward antimicrobial use and AMR; and the fourth explored practices related to AMU.

Both the baseline and endline surveys were administered by the same team of six trained enumerators drawn from the CAZAAP project, all of whom had completed data-collection and antimicrobial stewardship training delivered through the project. Retaining an identical enumerator team across both time points minimised inter-enumerator variability as a source of measured change. The questionnaire was digitised and deployed using the Kobo Collect tool, which incorporated skip logic and built-in validation constraints to standardise administration and reduce entry errors, and submissions were reviewed daily by the data manager for completeness, consistency, and logical accuracy, with feedback provided to the field team to resolve any issues. The identical questionnaire was administered at baseline and endline, with no additions, deletions, or changes to the wording of any knowledge, attitude, or practice item between time points, ensuring that observed differences reflect changes in respondents’ answers rather than changes in the instrument.

Knowledge items requiring correct understanding (definitions of antibiotics, AMR, antibiotic residues, and the withdrawal period) were scored against a predefined marking guide developed by the research team from WOAH reference definitions. A response was classified as correct only if it contained the essential elements specified in the guide; partial or inaccurate responses were classified as incorrect. Responses were scored independently by two reviewers (W.G. and V.C.), with any disagreements resolved by consensus, and the same marking guide was applied identically at baseline and endline. Because knowledge, attitude, and practice items were analysed individually rather than combined into summed composite scales, internal-consistency coefficients such as Cronbach’s alpha were not applicable; instrument validity was instead supported through content and face validity, with the questionnaire developed by the research team, reviewed by subject-matter experts in veterinary medicine and AMR, and pilot-tested among veterinary personnel in a non-study district (Kabwe) prior to administration.

### Qualitative data collection

Qualitative data were collected using KIIs with an interview guide to provide deeper insights into participants’ experiences, perceptions and practices. Informed consent was obtained from all participants prior to data collection. Separate consent was sought for audio recording of KIIs. Discussions and interviews were audio-recorded using digital voice recorders and phones. All recordings were transcribed verbatim and analysed using MAXQDA software. The interviewer was external to the veterinary services in the study districts and was not in a supervisory or regulatory relationship with any informant, which we considered likely to reduce social-desirability pressure during interviews. As a member of the CAZAAP project team, the interviewer held a prior interest in antimicrobial stewardship; to limit the influence of these assumptions on data generation, the interview guide used open, non-leading questions, informants were encouraged to describe their own experiences and to express disagreement, and analytic decisions were documented and discussed within the research team. Informants were aware that the interviewer was conducting the interviews as part of the CAZAAP project’s evaluation of antimicrobial stewardship.

### Data analysis

#### Quantitative data analyses

Data were entered into Microsoft Excel and analysed using R version 4. Descriptive statistics were used to summarize responses. Categorical variables were presented as frequencies and percentages.

This was a panel study in which the same individuals, linked by identifier, were surveyed at baseline and endline (49 complete pairs available at endline). Comparisons between respondents from Copperbelt and Lusaka provinces at each time point were conducted using Fisher’s Exact Test, owing to the small sample sizes and the presence of cells with expected frequencies less than five; these between-province comparisons are between-subjects. Within-cohort changes from baseline to endline are reported descriptively. Statistical significance was assessed at a 5% level (p < 0.05).

No formal *a priori* sample-size calculation was performed. The study sought to enrol all veterinary professionals and paraprofessionals serving the CAZAAP intervention districts, and the achieved samples (baseline n = 50; endline n = 49) therefore represent a near-complete enumeration of this finite and relatively small workforce rather than a sample sized to a predetermined effect. To characterise the resulting statistical sensitivity, a *post-hoc* sensitivity analysis was conducted. At a two-sided significance level of 0.05, the achieved sample provided approximately 80% power to detect only large differences in proportions: for the between-province comparisons (e.g., Copperbelt n = 33 versus Lusaka n = 16 at endline), the minimum detectable difference was on the order of 38–41 percentage points (from reference proportions of 30–50%), and for the overall baseline-to-endline comparison (n ≈ 50 per round) approximately 25–30 percentage points. As these estimates are based on the normal approximation, the exact power of Fisher’s exact test at these sample sizes would be lower still. Retrospective observed power was not reported, as it is a deterministic function of the observed p-value and provides no information beyond it. The study was thus adequately sensitive only to large effects and underpowered to detect small-to-moderate differences.

#### Qualitative data analyses

Qualitative data from key informant interviews were analyzed using MAXQDA software. Broad thematic areas, including knowledge, attitudes, and practices, were initially identified, followed by the development of more specific themes, including emergent themes arising from the data.

Qualitative changes in perceptions, barriers, and practices were analysed using endline data, including the exploration of participants’ reflections on changes in their practices and perceptions over the study period. Key thematic constructs were selected and triangulated with quantitative KAP survey findings to enhance interpretation and provide in-depth insights aligned with the study objectives.

Integration of the two strands followed a convergent mixed-methods logic. After separate analysis, findings were merged at the level of shared constructs (knowledge, attitudes, practices, and system-level drivers) using a joint display, in which each quantitative result was juxtaposed with the corresponding qualitative finding and assigned a fit category: convergence (agreement), divergence (disagreement), or expansion (one strand extending the other) and a meta-inference was derived. Where strands appeared discordant, we examined whether the discordance reflected genuinely conflicting evidence or differences in the construct, reference population, or measurement mode being captured.

### Ethical consideration

The study was approved by ERES (Ref 2023-June-020). Participation in this study was voluntary and unpaid. Written informed consent was obtained, and for the KIIs, recorded verbal consent was also obtained.

## Results

### Province-level baseline vs endline comparison: Copperbelt and Lusaka

The results presented below summarize the quantitative findings from the baseline and endline studies conducted on veterinary personnel in Copperbelt and Lusaka Provinces. These results highlight changes in knowledge, attitudes, and practices related to antimicrobial use and resistance over the study period. Detailed baseline and endline results for each province are provided in the **Supplementary Materials 1**. In addition, the full qualitative analysis reports are also included in the **Supplementary Materials** for further reference.

A total of 50 respondents participated in the baseline survey, comprising 34 respondents from Copperbelt Province and 16 from Lusaka Province. At endline, 49 respondents participated, including 33 from Copperbelt Province and 16 from Lusaka Province.

The study followed the same cohort of veterinary personnel across both time points; of the 50 participants enrolled at baseline, 49 were retained at endline. The demographic characteristics of the cohort at baseline and endline are presented in **Table 1**.

**Table 1:** Demographic characteristics of the study cohort at baseline and endline.

Characteristic	Baseline (N = 50)	Endline (N = 49)
Age (years)		
Mean (SD)	39 (10)	37 (9)
Median (Q1, Q3)	36 (29, 46)	35 (29, 43)
Min, Max	25, 64	26, 58
Years in practice		
Mean (SD)	11 (9)	10 (8)
Median (Q1, Q3)	9 (4, 17)	8 (4, 14)
Min, Max	1, 29	1, 30
Monthly income (ZMW)		
Mean (SD)	8, 558 (4, 442)	10, 359 (4, 979)
Median (Q1, Q3)	8, 000 (5, 600, 12, 000)	10, 000 (8, 000, 12, 000)
Min, Max	1, 300, 21, 400	2, 700, 26, 000

The mean age of the cohort was 39 years (SD ±10) at baseline and 37 years (SD ±9) at endline, with median ages of 36 and 35 years respectively. The age range remained broad (approximately 25–64 years at baseline and 26–58 years at endline), suggesting inclusion of both early-career and senior professionals.

Years in practice showed a similar profile, with mean experience of 11 years (SD ±9) at baseline and 10 years (SD ±8) at endline and median values of 9 and 8 years. The wide range (1–30 years) indicates substantial variability in experience levels, consistent with a moderately experienced cohort.

Monthly income increased over the study period. The mean income rose from ZMW 8, 558 (SD ±4, 442) at baseline to ZMW 10, 359 (SD ±4, 979) at endline, with median income increasing from ZMW 8, 000 to ZMW 10, 000. The income range also shifted upward, with the minimum increasing from ZMW 1, 300 to ZMW 2, 700 and the maximum from ZMW 21, 400 to ZMW 26, 000. As the same individuals were followed over time, this increase most plausibly reflects broader economic and inflationary trends over the study interval rather than a change in cohort composition.

Overall, the cohort comprised a moderately experienced veterinary workforce whose age and experience profile was broadly stable across the study, alongside a clear upward trend in income.

### Level of knowledge towards antibiotic use and stewardship

The results indicate generally high levels of awareness of antibiotics and antimicrobial resistance among respondents in both Copperbelt and Lusaka Provinces at baseline and endline (**Table 2**). At baseline, all respondents (100%) in both provinces reported knowing what antibiotics were, and this remained unchanged at endline. However, the proportion of respondents able to correctly define antibiotics improved over time, particularly in Copperbelt, increasing from 68% at baseline to 94% at endline, while Lusaka showed a smaller increase from 81% to 88%.

**Table 2:** Cross tabulation of knowledge characteristics by province.

Characteristic	Baseline (N = 50)			Endline (N = 49)		
	Copperbelt N = 34	Lusaka N = 16	p-value	Copperbelt N = 33	Lusaka N = 16	p-value
Do you know what Antibiotics/antimicrobials are	34 (100%)	16 (100%)	>0.9	33 (100%)	16 (100%)	>0.9
Correct Definition of Antibiotics/antimicrobials						
			0.2			0.6
Wrong explanation	7 (21%)	0 (0%)		2 (6.1%)	2 (13%)	
Partial correct	4 (12%)	3 (19%)		0 (0%)	0 (0%)	
Correct explanation	23 (68%)	13 (81%)		31 (94%)	14 (88%)	
Know what antibiotic/antimicrobial resistance is	32 (94%)	16 (100%)	>0.9	32 (97%)	16 (100%)	>0.9
Correct Definition of AMR						
			0.2			>0.9
Wrong explanation	2 (6.3%)	4 (25%)		7 (22%)	4 (25%)	
Partial correct	10 (31%)	5 (31%)		0 (0%)	0 (0%)	
Correct explanation	20 (63%)	7 (44%)		25 (78%)	12 (75%)	
Know what Antibiotic residues are	18 (53%)	12 (75%)	0.2	25 (76%)	15 (94%)	0.2
Correct Definition of Antibiotic residues						
			0.2			0.8
Wrong explanation	4 (22%)	0 (0%)		3 (12%)	3 (20%)	
Partial correct	7 (39%)	8 (67%)		1 (4.0%)	0 (0%)	
Correct explanation	7 (39%)	4 (33%)		21 (84%)	12 (80%)	
Know what the Withdrawal period is	34 (100%)	16 (100%)	>0.9	33 (100%)	16 (100%)	>0.9
Correct Definition of Withdrawal period						
			0.4			>0.9
Wrong explanation	5 (15%)	5 (31%)		3 (9.1%)	2 (13%)	
Partial correct	2 (5.9%)	1 (6.3%)		2 (6.1%)	0 (0%)	
Correct explanation	27 (79%)	10 (63%)		28 (85%)	14 (88%)	
AMR can cross between animals and humans						
			0.3			**0.004**
Strongly agree	25 (74%)	10 (63%)		28 (85%)	7 (44%)	
Agree	7 (21%)	5 (31%)		4 (12%)	7 (44%)	
Neutral	0 (0%)	0 (0%)		1 (3.0%)	0 (0%)	
Disagree	2 (5.9%)	0 (0%)		0 (0%)	1 (6.3%)	
Strongly disagree	0 (0%)	1 (6.3%)		0 (0%)	1 (6.3%)	
Know how to safely dispose of unused antimicrobials	27 (79%)	12 (75%)	0.7	28 (85%)	16 (100%)	0.2

Bold values indicate statistically significant differences between provinces (p < 0.05).

Knowledge of AMR was also high, with 94% of respondents in Copperbelt and 100% in Lusaka reporting awareness at baseline, increasing slightly to 97% and 100%, respectively, at endline. The proportion of respondents providing a correct definition of AMR improved in both provinces, rising from 63% to 78% in Copperbelt and from 44% to 75% in Lusaka. Despite this improvement, a notable proportion of respondents still provided incorrect definitions.

Awareness of antibiotic residues showed marked improvement, especially in Copperbelt, where knowledge increased from 53% at baseline to 76% at endline. Lusaka also demonstrated high awareness, increasing from 75% to 94%. Correspondingly, the proportion of correct definitions of antibiotic residues increased substantially in both provinces, reaching 84% in Copperbelt and 80% in Lusaka at endline.

All respondents in both provinces reported knowing what the withdrawal period was at both baseline and endline (100%). The proportion of correct definitions improved modestly, from 79% to 85% in Copperbelt and more substantially from 63% to 88% in Lusaka, indicating better understanding over time.

Regarding perceptions of AMR transmission between animals and humans, most respondents agreed or strongly agreed at both time points. However, a statistically significant difference between provinces was observed at endline (p = 0.004), with a higher proportion of respondents in Copperbelt strongly agreeing (85%) compared to Lusaka (44%), where responses were more distributed between “agree” and “strongly agree.”

Finally, knowledge of safe disposal of unused antimicrobials was relatively high and improved slightly over time, from 79% to 85% in Copperbelt and from 75% to 100% in Lusaka. Overall, while baseline awareness of key AMR concepts was high, the findings demonstrate notable improvements in depth of knowledge and correct understanding across both provinces over the study period.

### Attitude towards antibiotic use and stewardship

The results demonstrate generally positive attitudes toward antimicrobial stewardship across both provinces, with some notable shifts between baseline and endline and emerging differences between Copperbelt and Lusaka (**Table 3**).

**Table 3:** Cross tabulation of attitude characteristics by province.

Characteristic	Baseline (N = 50)			Endline (N = 49)		
	Copperbelt N = 34	Lusaka N = 16	p-value	Copperbelt N = 33	Lusaka N = 16	p-value
Good biosecurity/hygiene can reduce antibiotic use						
			0.4			**0.002**
Strongly agree	30 (88%)	15 (94%)		33 (100%)	11 (69%)	
Agree	3 (8.8%)	0 (0%)		0 (0%)	4 (25%)	
Neutral	0 (0%)	0 (0%)		0 (0%)	0 (0%)	
Disagree	1 (2.9%)	0 (0%)		0 (0%)	0 (0%)	
Strongly disagree	0 (0%)	1 (6.3%)		0 (0%)	1 (6.3%)	
Important to recommend biosecurity to farmers (daily practice)						
			>0.9			**0.008**
Strongly agree	33 (97%)	16 (100%)		30 (91%)	9 (56%)	
Agree	1 (2.9%)	0 (0%)		3 (9.1%)	6 (38%)	
Neutral	0 (0%)	0 (0%)		0 (0%)	0 (0%)	
Disagree	0 (0%)	0 (0%)		0 (0%)	0 (0%)	
Strongly disagree	0 (0%)	0 (0%)		0 (0%)	1 (6.3%)	
Important to train farmers on good management practices						
			>0.9			**0.034**
Strongly agree	32 (94%)	16 (100%)		32 (97%)	12 (75%)	
Agree	1 (2.9%)	0 (0%)		1 (3.0%)	3 (19%)	
Neutral	1 (2.9%)	0 (0%)		0 (0%)	0 (0%)	
Disagree	0 (0%)	0 (0%)		0 (0%)	0 (0%)	
Strongly disagree	0 (0%)	0 (0%)		0 (0%)	1 (6.3%)	
Prescription necessary before antibiotic sale	34 (100%)	16 (100%)		33 (100%)	16 (100%)	
Improper disposal of antimicrobials affects environment/health						
			>0.9			>0.9
No	0 (0%)	0 (0%)		0 (0%)	0 (0%)	
Yes	34 (100%)	16 (100%)		33 (100%)	16 (100%)	
Not sure	0 (0%)	0 (0%)		0 (0%)	0 (0%)	
AMR is an important problem for animal health						
			>0.9			**0.025**
Strongly agree	31 (91%)	16 (100%)		29 (88%)	9 (56%)	
Agree	2 (5.9%)	0 (0%)		4 (12%)	7 (44%)	
Neutral	1 (2.9%)	0 (0%)		0 (0%)	0 (0%)	
Disagree	0 (0%)	0 (0%)		0 (0%)	0 (0%)	
Strongly disagree	0 (0%)	0 (0%)		0 (0%)	0 (0%)	
AMR is an important problem for public health						
			0.8			**0.008**
Strongly agree	29 (85%)	16 (100%)		30 (91%)	9 (56%)	
Agree	3 (8.8%)	0 (0%)		3 (9.1%)	7 (44%)	
Neutral	1 (2.9%)	0 (0%)		0 (0%)	0 (0%)	
Disagree	0 (0%)	0 (0%)		0 (0%)	0 (0%)	
Strongly disagree	1 (2.9%)	0 (0%)		0 (0%)	0 (0%)	
Vaccinating broilers can reduce antibiotic use						
			0.6			0.067
Strongly agree	22 (65%)	12 (75%)		29 (88%)	10 (63%)	
Agree	9 (26%)	2 (13%)		4 (12%)	5 (31%)	
Neutral	1 (2.9%)	0 (0%)		0 (0%)	1 (6.3%)	
Disagree	2 (5.9%)	2 (13%)		0 (0%)	0 (0%)	
Strongly disagree	0 (0%)	0 (0%)		0 (0%)	0 (0%)	
Antibiotics given too often might stop working						
			0.4			**0.036**
Strongly agree	26 (76%)	12 (75%)		30 (91%)	10 (63%)	
Agree	7 (21%)	2 (13%)		3 (9.1%)	5 (31%)	
Neutral	0 (0%)	1 (6.3%)		0 (0%)	0 (0%)	
Disagree	1 (2.9%)	1 (6.3%)		0 (0%)	1 (6.3%)	
Strongly disagree	0 (0%)	0 (0%)		0 (0%)	0 (0%)	
The way antimicrobials are disposed of matters						
			>0.9			**0.010**
Strongly agree	30 (88%)	14 (88%)		29 (88%)	8 (50%)	
Agree	3 (8.8%)	2 (13%)		4 (12%)	8 (50%)	
Neutral	1 (2.9%)	0 (0%)		0 (0%)	0 (0%)	
Disagree	0 (0%)	0 (0%)		0 (0%)	0 (0%)	
Strongly disagree	0 (0%)	0 (0%)		0 (0%)	0 (0%)	

Bold values indicate statistically significant differences between provinces (p < 0.05).

At baseline, respondents in both provinces overwhelmingly agreed that good biosecurity and hygiene can reduce antibiotic use, with 88% in Copperbelt and 94% in Lusaka strongly agreeing. By endline, this strengthened further in Copperbelt (100% strongly agree), while Lusaka showed a shift toward less intense agreement (69% strongly agree, 25% agree), resulting in a statistically significant provincial difference (p = 0.002). A similar pattern was observed regarding the importance of recommending biosecurity to farmers: while near-universal strong agreement was reported at baseline in both provinces, this declined in Lusaka at endline (56% strongly agree, 38% agree), compared to consistently high levels in Copperbelt (91% strongly agree), yielding a significant difference (p = 0.008).

The importance of training farmers on good management practices remained highly endorsed across both provinces, although Lusaka again showed a slight decline in strong agreement at endline (75% strongly agree) compared to Copperbelt (97%), with a statistically significant difference (p = 0.034). Notably, all respondents in both provinces consistently agreed that a prescription is necessary before antibiotic sale and that improper disposal of antimicrobials negatively affects health and the environment, indicating strong consensus on these issues.

Perceptions of AMR as a critical issue were also high. At baseline, nearly all respondents strongly agreed that AMR was an important problem for both animal and public health. However, at endline, Lusaka respondents were less likely to strongly agree compared to Copperbelt, with more selecting “agree, “ resulting in significant differences for both animal health (p = 0.025) and public health (p = 0.008).

Attitudes toward preventive measures such as vaccination improved in Copperbelt, with strong agreement increasing from 65% to 88%, while Lusaka showed more moderate agreement at endline (63% strongly agree, 31% agree), though this difference was not statistically significant (p = 0.067). Similarly, recognition that overuse of antibiotics reduces their effectiveness increased in Copperbelt (91% strongly agree at endline) but was less pronounced in Lusaka (63% strongly agree), with a significant difference between provinces (p = 0.036).

Perceptions regarding antimicrobial disposal also diverged over time. While strong agreement that disposal practices matter remained high in Copperbelt (88%), Lusaka respondents shifted toward a more moderate stance (50% strongly agree, 50% agree), leading to a significant difference at endline (p = 0.010).

Overall, while attitudes toward AMR and stewardship remain positive, the results indicate a divergence between provinces over time, with Copperbelt generally maintaining or strengthening strong agreement, while Lusaka personnel show a shift toward more moderate positions.

### Practices on antibiotic use and stewardship

The findings indicate generally appropriate antimicrobial use practices among professionals, with some notable improvements over time, alongside persistent gaps in specific areas (**Table 4**).

**Table 4:** Cross tabulation of practice characteristics by province.

Characteristic	Baseline (N = 50)			Endline (N = 49)		
	Copperbelt N = 34	Lusaka N = 16	p-value	Copperbelt N = 33	Lusaka N = 16	p-value
Frequency of sending samples to lab for bacterial identification						
			0.079			<0.001
Never	9 (26%)	0 (0%)		15 (45%)	2 (13%)	
Rarely	13 (38%)	7 (44%)		14 (42%)	2 (13%)	
Sometimes	11 (32%)	8 (50%)		4 (12%)	8 (50%)	
Almost always	1 (2.9%)	1 (6.3%)		0 (0%)	3 (19%)	
Always	0 (0%)	0 (0%)		0 (0%)	1 (6.3%)	
Educate clients on observing antibiotic withdrawal period						
			>0.9			>0.9
No	0 (0%)	0 (0%)		0 (0%)	0 (0%)	
Yes	31 (91%)	15 (94%)		32 (97%)	15 (94%)	
Sometimes	3 (8.8%)	1 (6.3%)		1 (3.0%)	1 (6.3%)	
Frequency of using antibiotics for viral infections in broilers						
			>0.9			0.6
Never	1 (2.9%)	0 (0%)		9 (27%)	2 (13%)	
Rarely	5 (15%)	3 (19%)		4 (12%)	3 (19%)	
Sometimes	9 (26%)	4 (25%)		12 (36%)	6 (38%)	
Almost always	12 (35%)	5 (31%)		7 (21%)	3 (19%)	
Always	7 (21%)	4 (25%)		1 (3.0%)	2 (13%)	
Have a prescribed vaccination programme for broilers	25 (74%)	14 (88%)	0.5	27 (82%)	9 (56%)	0.086
Have treatment guidelines for antibiotic prescribing						
			0.091			0.4
No	32 (94%)	13 (81%)		25 (76%)	12 (75%)	
Yes	1 (2.9%)	3 (19%)		8 (24%)	3 (19%)	
Not sure	1 (2.9%)	0 (0%)		0 (0%)	1 (6.3%)	
Wear farm-specific clothing when visiting poultry farms						
			0.12			>0.9
No	11 (32%)	1 (6.3%)		6 (18%)	2 (13%)	
Yes	14 (41%)	10 (63%)		24 (73%)	12 (75%)	
Sometimes	9 (26%)	5 (31%)		3 (9.1%)	2 (13%)	
Use footbath when provided before entering poultry house	34 (100%)	16 (100%)	>0.9	33 (100%)	16 (100%)	>0.9
Prescribe antibiotics for disease prevention						
			>0.9			0.3
Yes, often	4 (12%)	1 (6.3%)		0 (0%)	0 (0%)	
Yes, sometimes	4 (12%)	2 (13%)		4 (12%)	0 (0%)	
Never	26 (76%)	13 (81%)		29 (88%)	16 (100%)	
Prescribe antibiotics in feed for growth promotion						
			>0.9			>0.9
Yes, often	0 (0%)	0 (0%)		0 (0%)	0 (0%)	
Yes, sometimes	3 (8.8%)	1 (6.3%)		1 (3.0%)	0 (0%)	
Never	31 (91%)	15 (94%)		32 (97%)	16 (100%)	
Sell antibiotics	8 (24%)	0 (0%)	**0.043**	6 (18%)	2 (13%)	>0.9
Write dosing regimen on antibiotic prescription						
			0.2			0.5
Yes, often	21 (62%)	11 (69%)		23 (70%)	13 (81%)	
Yes, sometimes	11 (32%)	2 (13%)		4 (12%)	2 (13%)	
Never	2 (5.9%)	3 (19%)		6 (18%)	1 (6.3%)	
Write withdrawal time on antibiotic prescription						
			0.2			0.3
Yes, often	22 (65%)	8 (50%)		26 (79%)	10 (63%)	
Yes, sometimes	1 (2.9%)	3 (19%)		2 (6.1%)	3 (19%)	
Never	11 (32%)	5 (31%)		5 (15%)	3 (19%)	

Bold values indicate statistically significant differences between provinces (p < 0.05).

Diagnostic practices revealed important gaps in both provinces. At baseline, most respondents reported rarely or sometimes sending samples for laboratory identification. By endline, this trend worsened in Copperbelt, with 45% reporting they never sent samples, while Lusaka showed some improvement, with increased reporting of “sometimes” and “almost always.” This difference was highly significant (p < 0.001), highlighting disparities in access to or utilization of diagnostic services.

Education of clients on observing antibiotic withdrawal periods was consistently high across both provinces, with over 90% of respondents reporting that they provide such guidance at both baseline and endline. This suggests strong adherence to recommended communication practices, with no significant differences observed between provinces.

Use of antibiotics for viral infections showed improvement over time, particularly in Copperbelt. At baseline, a substantial proportion of respondents reported frequent inappropriate use, with 35% “almost always” and 21% “always” prescribing antibiotics for viral infections. By endline, this decreased markedly, with increases in those reporting “never” (27%) and reductions in “always” (3%). Lusaka showed a more modest improvement, though inappropriate use remained present, with 13% still reporting “always” prescribing antibiotics for viral infections. These changes, however, were not statistically significant.

The proportion of respondents reporting the use of prescribed vaccination programmes for broilers increased in Copperbelt (74% to 82%) but declined in Lusaka (88% to 56%) at endline, suggesting possible inconsistencies in preventive practices across provinces, although this difference was not statistically significant (p = 0.086).

Access to treatment guidelines for antibiotic prescribing remained limited, although some improvement was observed. In Copperbelt, the proportion of respondents reporting access to guidelines increased from 2.9% at baseline to 24% at endline. Despite this, the majority in both provinces still reported lacking guidelines, reinforcing a key system gap.

Biosecurity practices improved over time, particularly regarding the use of farm-specific clothing. In Copperbelt, consistent use increased from 41% to 73%, while Lusaka also maintained relatively high compliance (63% to 75%). Use of footbaths was universally reported (100%) across both provinces at both time points, indicating strong adherence to this practice.

Encouragingly, the practice of prescribing antibiotics for disease prevention decreased over time, with the majority of respondents in both provinces reporting “never” prescribing for this purpose at endline (88% in Copperbelt and 100% in Lusaka). Similarly, the use of antibiotics for growth promotion was rare and declined further, with nearly all respondents reporting “never” at endline.

At baseline, a significantly higher proportion of respondents in Copperbelt reported selling antibiotics (24%) compared to Lusaka (0%) (p = 0.043). By endline, this difference was no longer significant, with reduced reporting in Copperbelt (18%) and a small increase in Lusaka (13%).

Prescription practices showed moderate improvement. The proportion of respondents who “often” write dosing regimens increased slightly in both provinces, while those who “never” do so decreased in Lusaka but increased in Copperbelt, indicating some inconsistency. Writing withdrawal periods on prescriptions improved in Copperbelt (65% to 79% “often”) but remained variable in Lusaka.

Overall, while there are clear improvements in several key antimicrobial stewardship practices, particularly reduced misuse for viral infections and decreased prophylactic use, important gaps remain. These include limited access to treatment guidelines, inconsistent prescription practices, and variability in preventive measures such as vaccination, highlighting areas for targeted intervention.

### Qualitative findings

Three key informant interviews were conducted with senior veterinary professionals selected to represent complementary roles across both study provinces: a District Coordinator/veterinarian in Chongwe District (Lusaka Province); a District Veterinary Officer in Ndola (Copperbelt Province); and a Senior Veterinary Officer for Copperbelt Province, based in Ndola. All three key informants were veterinary professionals. Interviews continued until no substantially new major themes emerged across informants; given the small number of senior key informants available within the project districts, the KIIs are presented as a focused explanatory strand complementing the quantitative survey rather than as a claim of full thematic saturation. A full analysis of the KIIs is presented in **Supplementary Materials 3**.

### Disconnect between knowledge and practice

Key informants consistently emphasized that although awareness of antimicrobial resistance among veterinary professionals has improved, this knowledge does not always translate into appropriate stewardship practices. Participants described situations in which veterinary personnel continued to prescribe antimicrobials empirically without diagnostic confirmation due to practical constraints, limited laboratory access and economic considerations.

“Among the professionals, we might think that we know more — but in reality … there are times when we claim as professionals that we know more, but in practice we are the ones who at times perpetuate the emergence or the transmission.” — KII-1, Chongwe District Coordinator

“I’m yet to see them practising that and advising lesser use of these antimicrobials.” — KII-2, Ndola District Veterinary Officer

### Farmer self-medication and informal antimicrobial access

A major theme emerging from interviews was the widespread practice of farmer self-medication. Informants reported that poultry farmers commonly obtained antibiotics directly from agrovet shops without prescriptions and frequently relied on peer recommendations or previous experience when administering antibiotics. Key informants additionally reported inappropriate dosing practices, failure to observe withdrawal periods, and routine prophylactic use of antibiotics among poultry farmers.

“Their neighbour said, I gave this and it’s working. And that is how withdrawal periods are not followed unless you emphasise it.” — KII-2, Ndola District Veterinary Officer

“I had a case of a farmer who came to say, my pigs died last time — so I’ve decided to buy this particular drug and just administer before they get sick.” — KII-3, Ndola Senior Veterinary Officer

“When you tell them that they shouldn’t give drugs … most of them will be shocked. It’s like they’ve never heard that there’s a withdrawal period and what it means.” — KII-2, Ndola District Veterinary Officer

### Weak regulatory enforcement

Participants identified weak regulatory enforcement as a major contributor to inappropriate antimicrobial use. Agrovet shops were frequently reported to be selling antibiotics without prescriptions and operating with limited regulation. Informants also highlighted poor monitoring of veterinary pharmaceutical quality, inappropriate drug storage conditions, and weak inspection systems.

“They want to sell as much and make their target. So they will just sell the farmer whatever the farmer needs.” — KII-3, Ndola Senior Veterinary Officer

“Usually they want to offload products and won’t bother to get prescriptions or ask for them … in short they want to make profit.” — KII-1, Chongwe District Coordinator

Informants also noted that veterinary drugs were entering the supply chain through poorly regulated outlets, including general shops:

“Even the farmers’ shops, they also stock these veterinary drugs.” — KII-3, Ndola Senior Veterinary Officer

### Limited diagnostic capacity

Limited access to laboratory diagnostics emerged as a major stewardship barrier. Most veterinary personnel relied on empirical treatment rather than laboratory-guided therapy. Participants described situations in which antibiotics were repeatedly switched following treatment failure without diagnostic investigation, potentially contributing to the emergence and spread of antimicrobial resistance.

“If they don’t get better in two days, they will change the drug … because the neighbour said this … they don’t even say what they were treating for.” — KII-2, Ndola District Veterinary Officer

“In cases of mastitis, farmers use all types of antibiotics, then you ask them why they’ve been changing antibiotics and they can’t give you reasons.” — KII-1, Chongwe District Coordinator

### Economic pressures and prescribing behaviour

Economic pressures were identified as important drivers of inappropriate antimicrobial use. Informants explained that both farmers and veterinary personnel often prioritized rapid disease management and financial survival over stewardship principles. The high cost of veterinary consultation and laboratory testing discouraged farmers from seeking professional services, contributing to self-medication practices.

“Due to pressure, farmers at times underdose to save money.” — KII-1, Chongwe District Coordinator

“Farmers do follow withdrawal periods, but … they are usually under pressure to offload their stock onto the market.” — KII-1, Chongwe District Coordinator

### Integration of quantitative and qualitative findings

The most prominent apparent divergence concerned knowledge. On the survey, nearly all respondents reported knowing what antibiotics and AMR were (94–100%), whereas key informants estimated functional AMR knowledge among professionals at only 40–60%. These figures are not aligned despite measuring the same construct. When the informants’ estimate is compared against this matched indicator rather than the awareness item, the two strands converge: both locate true understanding well below stated awareness. The residual gap is further explained by the informants estimating applied competence across the broader professional population rather than the CAZAAP-district intervention recipients surveyed.

Among the professionals, we might think that we know more — but in reality … there are times when we claim as professionals that we know more, but in practice we are the ones who at times perpetuate the emergence or the transmission. — Chongwe District Coordinator

The integrated reading is therefore that high headline awareness masks shallower applied knowledge a meta-inference that neither strand established alone.

The quantitative and qualitative findings converged strongly on practice gaps: limited laboratory submission, persistent empirical prescribing and treatment-failure-driven antibiotic switching, and limited access to treatment guidelines were independently evident in both datasets. The qualitative strand then expanded these findings by supplying the structural and governance mechanisms unregulated agrovet dispensing, weak prescription enforcement, cold-chain and storage failures, and the economic pressures on both farmers and professionals that the survey could only hint at through items such as antibiotic selling and guideline access. A final, instructive divergence arose on withdrawal periods: although over 90% of professionals reported educating clients, informants described farmers as “shocked” to learn that withdrawal periods exist, indicating that provider-reported education does not translate into farmer comprehension or adherence. Taken together, the integration supports the paper’s central claim that stewardship in Zambia’s poultry sector is constrained less by professional awareness than by applied competence and the structural environment in which professionals operate.

## Discussion

The findings from the KAP survey and Key Informant Interviews converged around several key themes related to antimicrobial resistance and antimicrobial use in Zambia’s poultry sector, highlighting both improvements in knowledge and persistent gaps in practice and system-level stewardship.

Overall, knowledge of antibiotics and AMR among professionals was high, with nearly all respondents reporting familiarity with antimicrobials and AMR across both provinces. There was a notable improvement in correct definitions of antibiotics, AMR, and antibiotic residues from baseline to endline, particularly in the Copperbelt Province. Despite this, qualitative findings revealed that knowledge remains inconsistent in depth and application. KII participants estimated AMR knowledge among professionals to range from 40% to 60%, and substantially lower among farmers (10–45%). These figures represent participant perceptions and estimates rather than calculated or measured values Importantly, informants emphasized a disconnect between theoretical knowledge and actual practice, noting that even trained professionals may contribute to inappropriate antimicrobial use.

This gap between awareness and accurate understanding is consistent with findings from other African countries, where knowledge of antimicrobial use and withdrawal periods has been shown to be limited, fragmented, or often superficial (Caudell et al., 2017; Sawadogo et al., 2023). This discrepancy between awareness and understanding was also more pronounced in relation to AMR and antibiotic residues. A limited understanding of what antibiotics are increases the likelihood of misuse, such as administering them inappropriately for viral infections and incorrect dosing, all of which are practices that accelerate the emergence of antimicrobial resistance (Hutchings et al., 2019; Kumar et al., 2021). Furthermore, poor comprehension of the withdrawal period poses a risk of antibiotic residues remaining in animal products like meat and eggs, which may lead to allergic reactions in consumers, disruption of gut microbiota, and the development of resistant bacteria in humans due to continuous low-level exposure (Nonga et al., 2010; Mund et al., 2017).

Although nearly all participants claimed to know what AMR was, only 63% in Copperbelt and 44% in Lusaka gave correct definitions. Similarly, livestock keepers in five African countries were generally aware of antibiotics but their understanding of AMR and its implications was limited (Caudell et al., 2020). These findings suggest that while respondents may be familiar with key terminology, their understanding of the technical and practical implications remains limited, which could hinder appropriate antimicrobial use and contribute to the risk of resistance development. These results highlight the urgent need for targeted education and training interventions to strengthen understanding, particularly among frontline animal health actors, to support antimicrobial stewardship efforts and mitigate the spread of AMR in Zambia.

Attitudes toward AMR were generally positive, with most respondents agreeing that AMR is an important issue for both animal and public health and that improved biosecurity and vaccination can reduce antibiotic use. The majority also supported prescription-only access to antibiotics. However, KII findings suggested that these positive attitudes are not consistently translated into practice, with systemic and behavioral barriers limiting effective stewardship. Additionally, messaging around AMR was identified as a challenge, with overly alarmist communication potentially reducing engagement among stakeholders.

The vast majority of professionals in both provinces strongly agreed that good farm biosecurity reduces antibiotic use, and they consistently emphasized the importance of recommending biosecurity and training farmers in proper farm management. Similarly, a study in Nigeria also emphasized the role of farmer training and veterinary recommendations in improving biosecurity (Kabeta et al., 2024). The recommendation of biosecurity measures to farmers is crucial for preventing the introduction and spread of infectious diseases within and between farms, thereby reducing the need to use antimicrobial contributes significantly to antimicrobial stewardship.

Survey results indicated some improvements in responsible practices, including reduced prophylactic antibiotic use and increased adherence to prescribing guidelines. For example, the proportion of professionals who reported never prescribing antibiotics for disease prevention increased, and the use of antibiotics for viral infections declined over time. However, laboratory-guided treatment remained limited, with most professionals rarely or never submitting samples for diagnostic testing. These findings were strongly supported by KII data, which highlighted widespread empirical treatment, frequent switching of antibiotics without diagnosis, and continued use of antibiotics based on prior experience or peer advice as laboratory access was also limited.

A major theme emerging from the KIIs was the widespread practice of self-medication among farmers. Farmers commonly obtained antibiotics directly from agrovet shops without prescriptions and relied on informal advice from peers or vendors. While professionals reported educating farmers on withdrawal periods in the survey, KII findings revealed that understanding and adherence among farmers remained poor. Practices such as underdosing, overdosing, prophylactic use, and failure to observe withdrawal periods were frequently reported, driven by economic pressures and limited access to veterinary services.

A universal agreement was observed on the necessity of prescriptions before selling antibiotics. Providing prescriptions to farmers for antimicrobial use is essential to ensure the responsible and appropriate use of antimicrobials. Prescriptions guarantee that antimicrobials are only used when clinically necessary, based on a proper diagnosis by a qualified veterinarian (Gray et al., 2021; Samuels et al., 2021). This reduces the risk of misuse such as incorrect dosing, unnecessary treatment, or use of critically important antimicrobials which can contribute to the development and spread of AMR. Prescriptions also support accurate record-keeping, monitoring of antimicrobial use, and adherence to withdrawal periods to protect food safety (Gray et al., 2021). Moreover, the prescription process offers an opportunity for veterinarians to educate farmers on correct administration, biosecurity, and alternative disease prevention strategies strengthening AMS. However, despite the study showing good attitude towards prescription writing, studies done here in Zambia have shown that farmers are still obtaining antimicrobials without prescriptions from the veterinarians (Mudenda et al., 2024). One major challenge is limited access to qualified veterinary personnel, especially in rural areas where veterinary services are scarce or completely absent (Caudell et al., 2020). As a result, farmers often rely on informal sources such as agro-vet shops, unlicensed drug vendors, or fellow farmers for advice and medications, bypassing the need for prescriptions (Caudell et al., 2020). Additionally, the cost of private veterinary consultations and diagnostics can be prohibitive, discouraging farmers from seeking professional guidance. Lastly, the weak regulatory enforcement further contributes to the problem, as antimicrobials are frequently sold over the counter without proper prescriptions (Mudenda et al., 2024). Complicating this issue is a lack of awareness among farmers about the importance of responsible antimicrobial use and the risks associated with self-medicating livestock, including the development of antimicrobial resistance (Mudenda et al., 2022; Mudenda et al., 2023; Mudenda et al., 2024). These barriers collectively undermine efforts to promote prudent use of antibiotics in animal health.

Both data sources highlighted critical systemic challenges. The KAP survey showed that most professionals lacked access to formal treatment guidelines, although some improvement was observed at endline. This was reinforced by KII findings, where all informants reported the absence of accessible, standardized veterinary treatment guidelines in practice. Additionally, agrovet shops were identified as the primary source of antimicrobials, often operating without strict regulatory oversight and driven by commercial incentives. Weak enforcement of prescription policies, poor drug storage practices, and inconsistent expiry date monitoring further contribute to inappropriate AMU.

Encouragingly, improvements in biosecurity-related practices were observed, including increased use of farm-specific protective clothing and high compliance with footbath use. Professionals also widely acknowledged the importance of farmer training and preventive measures. However, KII findings indicated that implementation at the farm level remains inconsistent, particularly among small-scale and backyard poultry producers, where resource limitations and knowledge gaps persist.

Experiences of treatment failure were commonly reported in the KIIs, often linked to inappropriate antibiotic use and lack of diagnostic support. Farmers frequently respond to treatment failure by switching antibiotics rather than seeking veterinary guidance, further exacerbating resistance risks. Although quantitative data did not directly measure treatment outcomes, the limited use of laboratory diagnostics and continued empirical prescribing observed in the survey supports this concern.

### Limitations

This study had several limitations. Most importantly, the pre–post design lacked a concurrent control group, and therefore could not distinguish changes potentially associated with the CAZAAP interventions from secular trends and other concurrent exposures. During the study period (2024–2025), Zambia was simultaneously implementing its multisectoral National Action Plan on Antimicrobial Resistance and related awareness activities, and AMR received sustained national and international attention. The observed changes in knowledge, attitudes, and practices therefore could not be causally attributed to the CAZAAP interventions specifically, and should be interpreted as changes observed over the study period rather than as intervention effects. The interval between some interventions’ delivery and the endline survey was relatively short, which could affect interpreting the observed changes. The absence of a comparison group also means that maturation and time-varying confounders cannot be excluded; for example, respondents’ mean monthly income rose over the study interval (from ZMW 8, 558 to ZMW 10, 359), a change most plausibly reflecting broader economic and inflationary trends rather than the interventions, but one that illustrates that the cohort’s circumstances were not static over the observation period. In addition, because the same questionnaire was administered to the same cohort at baseline and endline, repeated testing may itself have contributed to the observed changes through a familiarisation or testing effect, independent of any intervention or secular trend; as data collection and intervention delivery overlapped over the study period, such testing effects and the social-desirability considerations discussed below particularly at endline, after respondents had been exposed to stewardship training cannot be separated from any genuine change.

A further limitation is that all knowledge, attitude, and practice data were self-reported, with no direct observation of prescribing behaviour, no verification against dispensing or sales records, and no objective quantification of antimicrobial use. Self-reported practices are susceptible to social desirability bias, whereby respondents may over-report recommended stewardship behaviours and under-report inappropriate ones; this risk may be heightened at endline, when participants having received antimicrobial stewardship training were more likely to recognise the expected responses, potentially inflating the apparent improvement in practices. The convergent qualitative key informant interviews partly mitigate this limitation by triangulating the survey data: in several constructs the integrated analysis showed that stated awareness exceeded applied competence and that professional self-reports of improved practice coexisted with persistent inappropriate use within the wider farmer ecosystem (**Table 5**), indicating that self-report alone would have overstated stewardship. Nonetheless, triangulation cannot fully eliminate reporting bias, and the practice findings should be interpreted as self-reported behaviours rather than directly observed antimicrobial use.

**Table 5:** Joint display integrating quantitative (KAP survey) and qualitative (key informant interview) findings by construct, with fit category and meta-inference.

Construct/theme	Quantitative finding (KAP survey)	Qualitative finding (KIIs)	Fit	Meta-inference
**Awareness vs. correct understanding**	~100% report knowing what antibiotics/AMR are, but correct AMR definitions were only 44% (Lusaka) and 63% (Copperbelt) at baseline (rising to 75% and 78% at endline); correct residue definitions 33–39% at baseline.	Informants estimate functional AMR knowledge among professionals at 40–60%; “we claim … we know more, but in practice we are the ones who at times perpetuate the emergence.”	**Apparent divergence convergence on matched construct**	Headline awareness overstates applied knowledge; correct-definition rates, not the binary awareness items, are the valid comparator for the qualitative estimate.
**Attitudes toward stewardship**	Strongly positive (biosecurity reduces use, prescription-only access, AMR importance); Lusaka shifts toward more moderate agreement at endline.	Positive attitudes confirmed but do not translate into practice; “panic”-framed AMR messaging causes disengagement.	**Convergence + expansion**	Positive attitudes are necessary but insufficient; message framing is an actionable lever the survey did not surface.
**Diagnostic/laboratory use**	Most respondents rarely or never submit samples; Copperbelt 45% “never” at endline (p < 0.001).	Empirical treatment predominates; antibiotics switched on treatment failure without diagnostic confirmation; limited laboratory access.	**Convergence**	Low diagnostic use is robustly triangulated a real system gap, not a self-report artefact.
**Prophylactic/viral-infection use**	Reduced over time on professional self-report (less use for viral infections, less prophylaxis at endline).	Prophylaxis still common at farmer level (“administer before they get sick”); professionals improving but still observed to perpetuate misuse.	**Partial divergence**	Professional practice improved while the surrounding farmer ecosystem did not, limiting the community-level effect of professional gains.
**Treatment guideline acces**	Access limited but improving (Copperbelt 2.9% → 24%); majority in both provinces still lack guidelines.	No informant had access; only human-health guidelines were findable; veterinary guidelines reported as “almost done” but undisseminated.	**Convergence**	A confirmed, actionable system gap; the quantitative uptick reflects early/partial dissemination, consistent with the qualitative account.
**Withdrawal-period education vs. adherence**	Over 90% of professionals report educating clients on withdrawal periods.	Farmers described as “shocked” to learn withdrawal periods exist; adherence poor under market pressure.	**Divergence (provider report vs. farmer outcome)**	Reported education does not equate to farmer comprehension or adherence a transmission/efficacy gap.
**Drug access, regulation & economics**	24% (Copperbelt) report selling antibiotics; 100% endorse the necessity of a prescription.	Agrovets the dominant, largely unregulated access point; profit-driven dispensing without prescription; weak enforcement; economic pressure on farmers and professionals.	**Expansion**	The binding constraints are structural, not merely knowledge-based directly supporting the paper’s central argument.

Bold values indicate statistically significant differences between provinces (p < 0.05).

A further set of limitations concerns sample size and statistical power. The achieved samples (baseline n = 50; endline n = 49) represent a near-complete enumeration of the veterinary professionals and paraprofessionals available in the study districts rather than a sample sized to a predetermined effect, and the subgroups particularly Lusaka (n = 16) are correspondingly small. A sensitivity analysis indicated that the study was adequately powered to detect only large differences in proportions (on the order of 38–41 percentage points for between-province comparisons and 25–30 percentage points for the overall baseline-to-endline comparison, at 80% power and α = 0.05). Non-significant findings should therefore be interpreted as effects the study was underpowered to confirm rather than as evidence of no change, and the risk of Type II error for smaller trends is substantial. Between-group comparisons were conducted using Fisher’s exact test, which provides valid inference under sparse cell counts; nonetheless, significant differences arising from small subgroup cells remain statistically unstable and sensitive to a few respondents, and the many unadjusted item-level comparisons increase the possibility of false-positive findings. Provincial subgroup comparisons are accordingly presented as exploratory and hypothesis-generating and should not be over-interpreted.

### Implications for policy and practice

The patterns observed in this study point to several implications for strengthening antimicrobial stewardship in Zambia’s poultry sector, each of which follows from a specific gap identified here rather than from general principle. The persistent shortfall in access to treatment guidelines reported by the majority of professionals at endline and by every key informant suggests that finalising and systematically disseminating national veterinary standard treatment guidelines, and embedding them within routine practice and continuing professional development, would be a high-yield measure; the modest endline improvement in guideline access (Copperbelt, 2.9% to 24%) is consistent with dissemination having only begun during the study period. The low and largely unchanged use of laboratory diagnostics, together with the qualitative accounts of treatment-failure-driven antibiotic switching, indicates that gains in knowledge are unlikely to translate into prudent use unless accompanied by improved diagnostic access and functional AMR surveillance that captures treatment failure.

Because antimicrobials were widely available over the counter through a commercially driven and weakly regulated agrovet network, while professionals near-universally endorsed prescription-only access, our findings reinforce the case for strengthening the regulation and inspection of veterinary drug outlets and enforcing prescription requirements. The recurring disconnect between high awareness and applied competence further suggests that professional training should extend beyond knowledge transfer toward behaviour-change-oriented continuing professional development, while the finding that provider reported education on withdrawal periods did not correspond to farmer comprehension argues for farmer-centred education designed around uptake and retention rather than message delivery alone. These implications are mutually reinforcing and unlikely to succeed in isolation, highlighting the need for a coordinated One Health response spanning the animal, human, and environmental health sectors.

## Conclusion

In conclusion, this mixed-methods study assessed knowledge, attitudes, and practices related to antimicrobial stewardship among veterinary professionals and paraprofessionals in Zambia’s poultry sector across a period spanning a package of stewardship activities. Over the study period, awareness of AMR was consistently high, and improvements were observed in the depth of knowledge and in several stewardship practices, including reduced use of antibiotics for viral infections and decreased prophylactic use. However, important gaps persisted among these professionals notably limited use of laboratory diagnostics, inconsistent access to treatment guidelines, and variable prescribing documentation and the qualitative findings indicate that these are sustained by structural and governance constraints beyond individual knowledge. These patterns, consistent with antimicrobial-stewardship evidence from other low- and middle-income settings, suggest that strengthening stewardship in the veterinary workforce will require not only continued professional training that targets applied competence, but also improved diagnostic access, accessible treatment guidelines, and stronger regulatory enforcement, delivered within a coordinated One Health framework.

## Data Availability

The raw data supporting the conclusions of this article will be made available by the authors, without undue reservation.

## References

[B1] Apata D. F. (2009). Antibiotic resistance in poultry. Int. J. Poult. Sci. 8, 404–408. doi: 10.3923/ijps.2009.404.408

[B2] Caudell M. A. Dorado-Garcia A. Eckford S. Creese C. Byarugaba D. K. Afakye K. . (2020). Towards a bottom-up understanding of antimicrobial use and resistance on the farm: A knowledge, attitudes, and practices survey across livestock systems in five African countries. PloS One 15, 1–26. doi: 10.1371/journal.pone.0220274 31978098 PMC6980545

[B3] Caudell M. A. Quinlan M. B. Subbiah M. Call D. R. Roulette C. J. Roulette J. W. . (2017). Antimicrobial use and veterinary care among agro-pastoralists in Northern Tanzania. PloS One 12 (1), e0170328. doi: 10.1371/journal.pone.0170328 28125722 PMC5268417

[B4] Chilawa S. Mudenda S. Daka V. Chileshe M. Matafwali S. Chabalenge B. . (2023). Knowledge, attitudes, and practices of poultry farmers on antimicrobial use and resistance in Kitwe, Zambia: Implications on antimicrobial stewardship. Open J. Anim. Sci. 13, 60–81. doi: 10.4236/ojas.2023.131005

[B5] Government of the Republic of Zambia . (2017). Multisectoral National Action Plan on Antimicrobial Resistance Lusaka, Zambia: Government of the Republic of Zambia.

[B6] Government of the Republic of Zambia . (2018). Ministry of Fisheries and Livestock Main Report Republic of Zambia Ministry of Fisheries and Livestock Republic of Zambia Ministry of National Development Planning Lusaka, Zambia: Government of the Republic of Zambia.

[B7] Gray P. Jenner R. Norris J. Page S. (2021). Antimicrobial prescribing guidelines for poultry. Aust. Vet. J. 99 (6), 181–235. doi: 10.1111/avj.13034 33782952 PMC8251962

[B8] Hutchings M. Truman A. Wilkinson B. (2019). Antibiotics: Past, present and future. Curr. Opin. Microbiol. 51, 72–80. doi: 10.1016/j.mib.2019.10.008 31733401

[B9] Kabeta T. Tolosa T. Nagara A. Chantziaras I. Croubels S. Van Immerseel F. . (2024). Awareness of poultry farmers of interconnected health risks: A cross-sectional study on mycotoxins, biosecurity, and salmonellosis in Jimma, Ethiopia. Animals 14 (23), 3441. doi: 10.3390/ani14233441 39682406 PMC11639823

[B10] Kiambi S. Mwanza R. Sirma A. Czerniak C. Kimani T. Kabali E. . (2021). Understanding antimicrobial use contexts in the poultry sector: Challenges for small-scale layer farms in Kenya. Antibiotics 10, 1–16. doi: 10.3390/antibiotics10020106 33499334 PMC7911778

[B11] Kumar M. Sarma D. K. Shubham S. Kumawat M. Verma V. Nina P. B. . (2021). Futuristic non-antibiotic therapies to combat antibiotic resistance: A review. Front. Microbiol. 12. doi: 10.3389/fmicb.2021.609459 33574807 PMC7870489

[B12] McEwen S. A. Collignon P. J. (2018). Antimicrobial resistance: A one health perspective. Microbiol. Spectr. 6 (2), ARBA-0009-2017. doi: 10.1128/microbiolspec.arba-0009-2017 29600770 PMC11633550

[B13] Mudenda S. Bumbangi F. N. Yamba K. Munyeme M. Malama S. Mukosha M. . (2023). Drivers of antimicrobial resistance in layer poultry farming: Evidence from high prevalence of multidrug-resistant Escherichia coli and Enterococci in Zambia. Vet. World 16 (9), 1803–1814. doi: 10.14202/vetworld.2023.1803-1814 37859964 PMC10583887

[B14] Mudenda S. Malama S. Munyeme M. Hang’ombe B. M. Mainda G. Kapona O. . (2022). Awareness of antimicrobial resistance and associated factors among layer poultry farmers in Zambia: Implications for surveillance and antimicrobial stewardship programs. Antibiotics 11, 1–12. doi: 10.3390/antibiotics11030383 35326846 PMC8944564

[B15] Mudenda S. Mulenga K. M. Nyirongo R. Chabalenge B. Chileshe C. Daka V. . (2024). Non-prescription sale and dispensing of antibiotics for prophylaxis in broiler chickens in Lusaka District, Zambia: Findings and implications on one health. JAC-Antimicrob. Resist. 6 (3), dlae094. doi: 10.1093/jacamr/dlae094 38863561 PMC11166086

[B16] Mund M. D. Khan U. H. Tahir U. Mustafa B. E. Fayyaz A. (2017). Antimicrobial drug residues in poultry products and implications on public health: A review. Int. J. Food Prop. 20, 1433–1446. doi: 10.1080/10942912.2016.1212874 37339054

[B17] Nhung N. T. Chansiripornchai N. Carrique-Mas J. J. (2017). Antimicrobial resistance in bacterial poultry pathogens: A review. Front. Vet. Sci. 4. doi: 10.3389/fvets.2017.00126 28848739 PMC5554362

[B18] Nikaido H. (2009). Multidrug resistance in bacteria. Annu. Rev. Biochem. 78, 119–146. doi: 10.1146/annurev.biochem.78.082907.145923 19231985 PMC2839888

[B19] Nonga H. E. Simon C. Karimuribo E. D. Mdegela R. H. (2010). Assessment of antimicrobial usage and residues in commercial chicken eggs from smallholder poultry keepers in Morogoro Municipality, Tanzania. Zoonoses Public Health 57, 339–344. doi: 10.1111/j.1863-2378.2008.01226.x 19486498

[B20] Nowbuth A. A. Asombang A. W. Tazinkeng N. N. Makinde O. Y. Sheets L. R. (2023). Antimicrobial resistance from a one health perspective in Zambia: A systematic review. Antimicrob. Resist. Infect. Control 12, 1–12. doi: 10.1186/s13756-023-01224-0 36869351 PMC9982795

[B21] Peterson E. Kaur P. (2018). Antibiotic resistance mechanisms in bacteria: Relationships between resistance determinants of antibiotic producers, environmental bacteria, and clinical pathogens. Front. Microbiol. 9. doi: 10.3389/fmicb.2018.02928 30555448 PMC6283892

[B22] Samuels R. Qekwana D. N. Oguttu J. W. Odoi A. (2021). Antibiotic prescription practices and attitudes towards the use of antimicrobials among veterinarians in the City of Tshwane, South Africa. PeerJ 9, e10144. doi: 10.7717/peerj.10144 33520429 PMC7811296

[B23] Sawadogo A. Kagambèga A. Moodley A. Ouedraogo A. A. Barro N. Dione M. (2023). Knowledge, attitudes, and practices related to antibiotic use and antibiotic resistance among poultry farmers in urban and peri-urban areas of Ouagadougou, Burkina Faso. Antibiotics 12 (1), 133. doi: 10.3390/antibiotics12010133 36671334 PMC9854744

[B24] Simoneit C. Burow E. Tenhagen B. A. Käsbohrer A. (2015). Oral administration of antimicrobials increase antimicrobial resistance in E. coli from chicken - A systematic review. Prev. Vet. Med. 118, 1–7. doi: 10.1016/j.prevetmed.2014.11.010 25433717

[B25] Velazquez-Meza M. E. Galarde-López M. Carrillo-Quiróz B. Alpuche-Aranda C. M. (2022). Antimicrobial resistance: One health approach. Vet. World 15, 743–749. doi: 10.14202/vetworld.2022.743-749 35497962 PMC9047147

[B26] WHO . (2015). WHO Library Cataloguing-in-Publication Data Global Action Plan on Antimicrobial Resistance Geneva: World Health Organization, ISBN 9789241509763.

[B27] Zambia Statistics Agency (2022). 2022 Census of Population and Housing: Preliminary Report (Lusaka: Zambia Statistics Agency). Available online at: https://www.zamstats.gov.zm/wp-content/uploads/2023/12/2022-Census-of-Population-and-Housing-Preliminary.pd (Accessed May 7, 2026).

