# Supplementary material for: Bridging knowledge and practice gaps in antimicrobial stewardship among veterinary professionals in Zambia’s poultry sector: a mixed-methods pre–post intervention study
Source: Front Antibiot. 2026 Jul 30;5:1898867. doi: 10.3389/frabi.2026.1898867 (PMC13469284; doi:10.3389/frabi.2026.1898867)
Supplement: Supplementary file 1 [file Table1.docx]

Professional Baseline Endline Analysis

William Ngosa

2026-04-16

# Baseline results

| **Characteristic** | **N = 50** |
| --- | --- |
| age |  |
| Mean (SD) | 39 (10) |
| Median (Q1, Q3) | 36 (29, 46) |
| Min, Max | 25, 64 |
| practiceyears |  |
| Mean (SD) | 11 (9) |
| Median (Q1, Q3) | 9 (4, 17) |
| Min, Max | 1, 29 |
| monthlyincome |  |
| Mean (SD) | 8,558 (4,442) |
| Median (Q1, Q3) | 8,000 (5,600, 12,000) |
| Min, Max | 1,300, 21,400 |

# Knowledge

| **Characteristic** | **Copperbelt**  N = 34^1^ | **Lusaka**  N = 16^1^ | **p-value**^2^ |
| --- | --- | --- | --- |
| Do you know what Antibiotics/antimicrobials are | 34 (100%) | 16 (100%) | >0.9 |
| Correct Definition of Antibiotics/antimicrobials |  |  | 0.2 |
| Wrong explanation | 7 (21%) | 0 (0%) |  |
| Partial correct | 4 (12%) | 3 (19%) |  |
| Correct explanation | 23 (68%) | 13 (81%) |  |
| Know what is antibiotic resistance or antimicrobial resistanc | 32 (94%) | 16 (100%) | >0.9 |
| Correct Definition of antibiotic resistance or antimicrobial resistanc |  |  | 0.2 |
| Wrong explanation | 2 (6.3%) | 4 (25%) |  |
| Partial correct | 10 (31%) | 5 (31%) |  |
| Correct explanation | 20 (63%) | 7 (44%) |  |
| Unknown | 2 | 0 |  |
| Know what Antibiotic residues are | 18 (53%) | 12 (75%) | 0.2 |
| Correct Definition of Antibiotic residues |  |  | 0.2 |
| Wrong explanation | 4 (22%) | 0 (0%) |  |
| Partial correct | 7 (39%) | 8 (67%) |  |
| Correct explanation | 7 (39%) | 4 (33%) |  |
| Unknown | 16 | 4 |  |
| know what the Withdrawal period is | 34 (100%) | 16 (100%) | >0.9 |
| Correct Definition of Withdrawal period |  |  | 0.4 |
| Wrong explanation | 5 (15%) | 5 (31%) |  |
| Partial correct | 2 (5.9%) | 1 (6.3%) |  |
| Correct explanation | 27 (79%) | 10 (63%) |  |
| Can antibiotic resistance or antimicrobial resistance cross between animals and humans |  |  | 0.3 |
| Strongly agree | 25 (74%) | 10 (63%) |  |
| Agree | 7 (21%) | 5 (31%) |  |
| Netural | 0 (0%) | 0 (0%) |  |
| Disagree | 2 (5.9%) | 0 (0%) |  |
| Strongly disagree | 0 (0%) | 1 (6.3%) |  |
| Do you know how to safely dispose of expired left over or un-used antimicrobials | 27 (79%) | 12 (75%) | 0.7 |
| ^1^n (%) | | | |
| ^2^Fisher's exact test | | | |

# Attitude

| **Characteristic** | **Copperbelt**  N = 34^1^ | **Lusaka**  N = 16^1^ | **p-value**^2^ |
| --- | --- | --- | --- |
| Good farm biosecurity and hygiene can reduce the use of antibiotics on the poultry farm |  |  | 0.4 |
| Strongly agree | 30 (88%) | 15 (94%) |  |
| Agree | 3 (8.8%) | 0 (0%) |  |
| Netural | 0 (0%) | 0 (0%) |  |
| Disagree | 1 (2.9%) | 0 (0%) |  |
| Strongly disagree | 0 (0%) | 1 (6.3%) |  |
| In my daily practice, it is important to recommend good biosecurity to the poultry farmers |  |  | >0.9 |
| Strongly agree | 33 (97%) | 16 (100%) |  |
| Agree | 1 (2.9%) | 0 (0%) |  |
| Netural | 0 (0%) | 0 (0%) |  |
| Disagree | 0 (0%) | 0 (0%) |  |
| Strongly disagree | 0 (0%) | 0 (0%) |  |
| It is important to train farmers on good farm management practices |  |  | >0.9 |
| Strongly agree | 32 (94%) | 16 (100%) |  |
| Agree | 1 (2.9%) | 0 (0%) |  |
| Netural | 1 (2.9%) | 0 (0%) |  |
| Disagree | 0 (0%) | 0 (0%) |  |
| Strongly disagree | 0 (0%) | 0 (0%) |  |
| Do you think that a prescription is necessary before sale of an antibiotic to a farmer | 34 (100%) | 16 (100%) |  |
| Can improper disposal of un-used and expired antimicrobials affect the environment and health |  |  | >0.9 |
| No | 0 (0%) | 0 (0%) |  |
| Yes | 34 (100%) | 16 (100%) |  |
| Not sure | 0 (0%) | 0 (0%) |  |
| Antibiotic resistance (or antimicrobial resistance) is an important problem for animal health. |  |  | >0.9 |
| Strongly agree | 31 (91%) | 16 (100%) |  |
| Agree | 2 (5.9%) | 0 (0%) |  |
| Netural | 1 (2.9%) | 0 (0%) |  |
| Disagree | 0 (0%) | 0 (0%) |  |
| Strongly disagree | 0 (0%) | 0 (0%) |  |
| Antibiotic resistance (or antimicrobial resistance) is an important problem for public health |  |  | 0.8 |
| Strongly agree | 29 (85%) | 16 (100%) |  |
| Agree | 3 (8.8%) | 0 (0%) |  |
| Netural | 1 (2.9%) | 0 (0%) |  |
| Disagree | 0 (0%) | 0 (0%) |  |
| Strongly disagree | 1 (2.9%) | 0 (0%) |  |
| Vaccinating broilers can reduce the need to use antibiotics |  |  | 0.6 |
| Strongly agree | 22 (65%) | 12 (75%) |  |
| Agree | 9 (26%) | 2 (13%) |  |
| Netural | 1 (2.9%) | 0 (0%) |  |
| Disagree | 2 (5.9%) | 2 (13%) |  |
| Strongly disagree | 0 (0%) | 0 (0%) |  |
| If antibiotics (or antimicrobials) are given too often, they might stop working |  |  | 0.4 |
| Strongly agree | 26 (76%) | 12 (75%) |  |
| Agree | 7 (21%) | 2 (13%) |  |
| Netural | 0 (0%) | 1 (6.3%) |  |
| Disagree | 1 (2.9%) | 1 (6.3%) |  |
| Strongly disagree | 0 (0%) | 0 (0%) |  |
| The way antimicrobials or antibiotics are disposed of matters |  |  | >0.9 |
| Strongly agree | 30 (88%) | 14 (88%) |  |
| Agree | 3 (8.8%) | 2 (13%) |  |
| Netural | 1 (2.9%) | 0 (0%) |  |
| Disagree | 0 (0%) | 0 (0%) |  |
| Strongly disagree | 0 (0%) | 0 (0%) |  |
| How often do you take and send samples to a laboratory for bacterial identification |  |  | 0.079 |
| Never | 9 (26%) | 0 (0%) |  |
| Rarely | 13 (38%) | 7 (44%) |  |
| Sometimes | 11 (32%) | 8 (50%) |  |
| Almost always | 1 (2.9%) | 1 (6.3%) |  |
| Alwyas | 0 (0%) | 0 (0%) |  |
| ^1^n (%) | | | |
| ^2^Fisher's exact test | | | |

# Practice

| **Characteristic** | **Copperbelt**  N = 34^1^ | **Lusaka**  N = 16^1^ | **p-value**^2^ |
| --- | --- | --- | --- |
| Do you always educate your clients about the need to observe the withdrawal period of antibiotics during prescribing or during treatment |  |  | >0.9 |
| No | 0 (0%) | 0 (0%) |  |
| Yes | 31 (91%) | 15 (94%) |  |
| Sometimes | 3 (8.8%) | 1 (6.3%) |  |
| In your practice, how frequently do you use antibiotics to treat or prevent viral infections in broilers |  |  | >0.9 |
| Never | 1 (2.9%) | 0 (0%) |  |
| Rarely | 5 (15%) | 3 (19%) |  |
| Sometimes | 9 (26%) | 4 (25%) |  |
| Almost always | 12 (35%) | 5 (31%) |  |
| Alwyas | 7 (21%) | 4 (25%) |  |
| Do you have a prescribed vaccination programme for the broiler birds which you recommend to farmers | 25 (74%) | 14 (88%) | 0.5 |
| Do you have any treatment guidelines that you use when prescribing the antibiotics to use for the different diseases and conditions |  |  | 0.091 |
| No | 32 (94%) | 13 (81%) |  |
| Yes | 1 (2.9%) | 3 (19%) |  |
| Not sure | 1 (2.9%) | 0 (0%) |  |
| Do you wear farm-specific clothing when you visit poultry farms |  |  | 0.12 |
| No | 11 (32%) | 1 (6.3%) |  |
| Yes | 14 (41%) | 10 (63%) |  |
| Sometimes | 9 (26%) | 5 (31%) |  |
| Do you use the footbath when it is provided before entry into the poultry house |  |  | >0.9 |
| No | 0 (0%) | 0 (0%) |  |
| Yes | 34 (100%) | 16 (100%) |  |
| Sometimes | 0 (0%) | 0 (0%) |  |
| Do you prescribe antibiotics for disease prevention |  |  | >0.9 |
| Yes, often | 4 (12%) | 1 (6.3%) |  |
| Yes, sometimes | 4 (12%) | 2 (13%) |  |
| Never | 26 (76%) | 13 (81%) |  |
| Do you prescribe antibiotics to be mixed in feed to boost the growth of broilers |  |  | >0.9 |
| Yes, often | 0 (0%) | 0 (0%) |  |
| Yes, sometimes | 3 (8.8%) | 1 (6.3%) |  |
| Never | 31 (91%) | 15 (94%) |  |
| Do you sell antibiotics | 8 (24%) | 0 (0%) | 0.043 |
| When you prescribe an antibiotic, do you write the dosing regimen on your prescription |  |  | 0.2 |
| Yes, often | 21 (62%) | 11 (69%) |  |
| Yes, sometimes | 11 (32%) | 2 (13%) |  |
| Never | 2 (5.9%) | 3 (19%) |  |
| When you prescribe an antibiotic, do you write the withdrawal time to be complied with |  |  | 0.2 |
| Yes, often | 22 (65%) | 8 (50%) |  |
| Yes, sometimes | 1 (2.9%) | 3 (19%) |  |
| Never | 11 (32%) | 5 (31%) |  |
| ^1^n (%) | | | |
| ^2^Fisher's exact test | | | |

# Endline results

| **Characteristic** | **N = 49** |
| --- | --- |
| age |  |
| Mean (SD) | 37 (9) |
| Median (Q1, Q3) | 35 (29, 43) |
| Min, Max | 26, 58 |
| practiceyears |  |
| Mean (SD) | 10 (8) |
| Median (Q1, Q3) | 8 (4, 14) |
| Min, Max | 1, 30 |
| monthlyincome |  |
| Mean (SD) | 10,359 (4,979) |
| Median (Q1, Q3) | 10,000 (8,000, 12,000) |
| Min, Max | 2,700, 26,000 |

# Knowledge

| **Characteristic** | **Copperbelt**  N = 33^1^ | **Lusaka**  N = 16^1^ | **p-value**^2^ |
| --- | --- | --- | --- |
| Do you know what Antibiotics/antimicrobials are | 33 (100%) | 16 (100%) | >0.9 |
| Correct Definition of Antibiotics/antimicrobials |  |  | 0.6 |
| Wrong explanation | 2 (6.1%) | 2 (13%) |  |
| Partial correct | 0 (0%) | 0 (0%) |  |
| Correct explanation | 31 (94%) | 14 (88%) |  |
| Know what is antibiotic resistance or antimicrobial resistanc | 32 (97%) | 16 (100%) | >0.9 |
| Correct Definition of antibiotic resistance or antimicrobial resistanc |  |  | >0.9 |
| Wrong explanation | 7 (22%) | 4 (25%) |  |
| Partial correct | 0 (0%) | 0 (0%) |  |
| Correct explanation | 25 (78%) | 12 (75%) |  |
| Unknown | 1 | 0 |  |
| Know what Antibiotic residues are | 25 (76%) | 15 (94%) | 0.2 |
| Correct Definition of Antibiotic residues |  |  | 0.8 |
| Wrong explanation | 3 (12%) | 3 (20%) |  |
| Partial correct | 1 (4.0%) | 0 (0%) |  |
| Correct explanation | 21 (84%) | 12 (80%) |  |
| Unknown | 8 | 1 |  |
| know what the Withdrawal period is | 33 (100%) | 16 (100%) | >0.9 |
| Correct Definition of Withdrawal period |  |  | >0.9 |
| Wrong explanation | 3 (9.1%) | 2 (13%) |  |
| Partial correct | 2 (6.1%) | 0 (0%) |  |
| Correct explanation | 28 (85%) | 14 (88%) |  |
| Can antibiotic resistance or antimicrobial resistance cross between animals and humans |  |  | 0.004 |
| Strongly agree | 28 (85%) | 7 (44%) |  |
| Agree | 4 (12%) | 7 (44%) |  |
| Netural | 1 (3.0%) | 0 (0%) |  |
| Disagree | 0 (0%) | 1 (6.3%) |  |
| Strongly disagree | 0 (0%) | 1 (6.3%) |  |
| Do you know how to safely dispose of expired left over or un-used antimicrobials | 28 (85%) | 16 (100%) | 0.2 |
| ^1^n (%) | | | |
| ^2^Fisher's exact test | | | |

# Attitude

| **Characteristic** | **Copperbelt**  N = 33^1^ | **Lusaka**  N = 16^1^ | **p-value**^2^ |
| --- | --- | --- | --- |
| Good farm biosecurity and hygiene can reduce the use of antibiotics on the poultry farm |  |  | 0.002 |
| Strongly agree | 33 (100%) | 11 (69%) |  |
| Agree | 0 (0%) | 4 (25%) |  |
| Netural | 0 (0%) | 0 (0%) |  |
| Disagree | 0 (0%) | 0 (0%) |  |
| Strongly disagree | 0 (0%) | 1 (6.3%) |  |
| In my daily practice, it is important to recommend good biosecurity to the poultry farmers |  |  | 0.008 |
| Strongly agree | 30 (91%) | 9 (56%) |  |
| Agree | 3 (9.1%) | 6 (38%) |  |
| Netural | 0 (0%) | 0 (0%) |  |
| Disagree | 0 (0%) | 0 (0%) |  |
| Strongly disagree | 0 (0%) | 1 (6.3%) |  |
| It is important to train farmers on good farm management practices |  |  | 0.034 |
| Strongly agree | 32 (97%) | 12 (75%) |  |
| Agree | 1 (3.0%) | 3 (19%) |  |
| Netural | 0 (0%) | 0 (0%) |  |
| Disagree | 0 (0%) | 0 (0%) |  |
| Strongly disagree | 0 (0%) | 1 (6.3%) |  |
| Do you think that a prescription is necessary before sale of an antibiotic to a farmer | 33 (100%) | 16 (100%) |  |
| Can improper disposal of un-used and expired antimicrobials affect the environment and health |  |  | >0.9 |
| No | 0 (0%) | 0 (0%) |  |
| Yes | 33 (100%) | 16 (100%) |  |
| Not sure | 0 (0%) | 0 (0%) |  |
| Antibiotic resistance (or antimicrobial resistance) is an important problem for animal health. |  |  | 0.025 |
| Strongly agree | 29 (88%) | 9 (56%) |  |
| Agree | 4 (12%) | 7 (44%) |  |
| Netural | 0 (0%) | 0 (0%) |  |
| Disagree | 0 (0%) | 0 (0%) |  |
| Strongly disagree | 0 (0%) | 0 (0%) |  |
| Antibiotic resistance (or antimicrobial resistance) is an important problem for public health |  |  | 0.008 |
| Strongly agree | 30 (91%) | 9 (56%) |  |
| Agree | 3 (9.1%) | 7 (44%) |  |
| Netural | 0 (0%) | 0 (0%) |  |
| Disagree | 0 (0%) | 0 (0%) |  |
| Strongly disagree | 0 (0%) | 0 (0%) |  |
| Vaccinating broilers can reduce the need to use antibiotics |  |  | 0.067 |
| Strongly agree | 29 (88%) | 10 (63%) |  |
| Agree | 4 (12%) | 5 (31%) |  |
| Netural | 0 (0%) | 1 (6.3%) |  |
| Disagree | 0 (0%) | 0 (0%) |  |
| Strongly disagree | 0 (0%) | 0 (0%) |  |
| If antibiotics (or antimicrobials) are given too often, they might stop working |  |  | 0.036 |
| Strongly agree | 30 (91%) | 10 (63%) |  |
| Agree | 3 (9.1%) | 5 (31%) |  |
| Netural | 0 (0%) | 0 (0%) |  |
| Disagree | 0 (0%) | 1 (6.3%) |  |
| Strongly disagree | 0 (0%) | 0 (0%) |  |
| The way antimicrobials or antibiotics are disposed of matters |  |  | 0.010 |
| Strongly agree | 29 (88%) | 8 (50%) |  |
| Agree | 4 (12%) | 8 (50%) |  |
| Netural | 0 (0%) | 0 (0%) |  |
| Disagree | 0 (0%) | 0 (0%) |  |
| Strongly disagree | 0 (0%) | 0 (0%) |  |
| How often do you take and send samples to a laboratory for bacterial identification |  |  | <0.001 |
| Never | 15 (45%) | 2 (13%) |  |
| Rarely | 14 (42%) | 2 (13%) |  |
| Sometimes | 4 (12%) | 8 (50%) |  |
| Almost always | 0 (0%) | 3 (19%) |  |
| Alwyas | 0 (0%) | 1 (6.3%) |  |
| ^1^n (%) | | | |
| ^2^Fisher's exact test | | | |

# Practice

| **Characteristic** | **Copperbelt**  N = 33^1^ | **Lusaka**  N = 16^1^ | **p-value**^2^ |
| --- | --- | --- | --- |
| Do you always educate your clients about the need to observe the withdrawal period of antibiotics during prescribing or during treatment |  |  | >0.9 |
| No | 0 (0%) | 0 (0%) |  |
| Yes | 32 (97%) | 15 (94%) |  |
| Sometimes | 1 (3.0%) | 1 (6.3%) |  |
| In your practice, how frequently do you use antibiotics to treat or prevent viral infections in broilers |  |  | 0.6 |
| Never | 9 (27%) | 2 (13%) |  |
| Rarely | 4 (12%) | 3 (19%) |  |
| Sometimes | 12 (36%) | 6 (38%) |  |
| Almost always | 7 (21%) | 3 (19%) |  |
| Alwyas | 1 (3.0%) | 2 (13%) |  |
| Do you have a prescribed vaccination programme for the broiler birds which you recommend to farmers | 27 (82%) | 9 (56%) | 0.086 |
| Do you have any treatment guidelines that you use when prescribing the antibiotics to use for the different diseases and conditions |  |  | 0.4 |
| No | 25 (76%) | 12 (75%) |  |
| Yes | 8 (24%) | 3 (19%) |  |
| Not sure | 0 (0%) | 1 (6.3%) |  |
| Do you wear farm-specific clothing when you visit poultry farms |  |  | >0.9 |
| No | 6 (18%) | 2 (13%) |  |
| Yes | 24 (73%) | 12 (75%) |  |
| Sometimes | 3 (9.1%) | 2 (13%) |  |
| Do you use the footbath when it is provided before entry into the poultry house |  |  | >0.9 |
| No | 0 (0%) | 0 (0%) |  |
| Yes | 33 (100%) | 16 (100%) |  |
| Sometimes | 0 (0%) | 0 (0%) |  |
| Do you prescribe antibiotics for disease prevention |  |  | 0.3 |
| Yes, often | 0 (0%) | 0 (0%) |  |
| Yes, sometimes | 4 (12%) | 0 (0%) |  |
| Never | 29 (88%) | 16 (100%) |  |
| Do you prescribe antibiotics to be mixed in feed to boost the growth of broilers |  |  | >0.9 |
| Yes, often | 0 (0%) | 0 (0%) |  |
| Yes, sometimes | 1 (3.0%) | 0 (0%) |  |
| Never | 32 (97%) | 16 (100%) |  |
| Do you sell antibiotics | 6 (18%) | 2 (13%) | >0.9 |
| When you prescribe an antibiotic, do you write the dosing regimen on your prescription |  |  | 0.5 |
| Yes, often | 23 (70%) | 13 (81%) |  |
| Yes, sometimes | 4 (12%) | 2 (13%) |  |
| Never | 6 (18%) | 1 (6.3%) |  |
| When you prescribe an antibiotic, do you write the withdrawal time to be complied with |  |  | 0.3 |
| Yes, often | 26 (79%) | 10 (63%) |  |
| Yes, sometimes | 2 (6.1%) | 3 (19%) |  |
| Never | 5 (15%) | 3 (19%) |  |
| ^1^n (%) | | | |
| ^2^Fisher's exact test | | | |
