# Supplementary material for: Bridging knowledge and practice gaps in antimicrobial stewardship among veterinary professionals in Zambia’s poultry sector: a mixed-methods pre–post intervention study
Source: Front Antibiot. 2026 Jul 30;5:1898867. doi: 10.3389/frabi.2026.1898867 (PMC13469284; doi:10.3389/frabi.2026.1898867)
Supplement: Supplementary file 2 [file Table2.docx]

**CAAZAP PROJECT**

**Key Informant Interview Analysis Report**

Antimicrobial Use & Resistance Among Poultry Sector Professionals

Chongwe District (Lusaka Province) & Ndola (Copperbelt Province) — April 2026

| **Study** | CAAZAP (Community Approaches to Antimicrobial Stewardship in Animal Production) |
| --- | --- |
| **Method** | Key Informant Interviews (KII) |
| **Number of interviews** | 3 (Chongwe District Coordinator; Ndola DVO; Ndola Senior Veterinary Officer, Copperbelt Province) |
| **Interviewer** | Chikwanda Chileshe |
| **Date** | April 2026 |
| **Geographic scope** | Chongwe District, Lusaka Province; Ndola, Copperbelt Province |
| **Key themes** | Drug supply; Farmer practices; Storage & expiry compliance; Record keeping; Treatment failure; AMR knowledge; Training; Interventions |

# 1. Background and Context

This report presents findings from three Key Informant Interviews (KIIs) conducted as part of the CAAZAP (Community Approaches to Antimicrobial Stewardship in Animal Production) project. The interviews were conducted by Chikwanda Chileshe with senior veterinary professionals serving in Chongwe District (Lusaka Province) and Ndola (Copperbelt Province). The purpose of the KIIs was to explore knowledge, attitudes, and perceptions regarding antimicrobial use (AMU) and antimicrobial resistance (AMR) among livestock professionals and the farmers they serve.

The three respondents occupied different roles in the animal health system: district-level coordination and extension (Chongwe District Coordinator, Dr Francis Mwanza); district veterinary oversight (Ndola DVO); and provincial senior veterinary operations (Ndola Senior Veterinary Officer for Copperbelt Province, Dr Caroline). Together their perspectives capture experiences from both major provinces of the CAAZAP study area.

# 2. Participant Profiles

The table below summarises the key informants, their locations, and their self-assessed knowledge estimates for AMR awareness among veterinary professionals and farmers in their districts.

| **Participant / Role** | **Location** | **AMR Knowledge Est.** | **Farmers' Knowledge Est.** |
| --- | --- | --- | --- |
| **Dr Francis Mwanza — District Coordinator (Vet)** | Chongwe, Lusaka | Vets: ~60% | Farmers: <50% |
| **Anonymous — District Veterinary Officer (DVO)** | Ndola, Copperbelt | Vets: ~51% | Farmers: ~45% |
| **Dr Caroline — Senior Veterinary Officer, Copperbelt Province** | Ndola, Copperbelt | Vets: ~40% | Farmers: ~10% |

The strikingly wide range in self-assessed knowledge estimates — from 10% to 60% for professionals and 10% to <50% for farmers — itself reflects inconsistent exposure to AMR education and the absence of a standardised national training programme. The most experienced respondent (Ndola Senior VO) was the most conservative in her estimates, noting that professionals' practised behaviour often lags behind their stated knowledge.

# 3. Thematic Findings

Seven cross-cutting themes emerged consistently across all three interviews. Each is presented below with supporting evidence from the transcripts.

## 3.1 Drug Supply and the Role of Agrovet Shops

Across all three sites, agrovet shops were identified as the primary — and frequently unregulated — point of access for antimicrobials. Key drugs in circulation include oxytetracyclines (the most widely mentioned across all interviews), penicillin/penstrep, sulfonamides (sulfadimidine/trimethoprim combinations), enrofloxacin, and amprolium as a coccidiostat. In Ndola, tiamulin and parvaquone (for East Coast Fever-endemic areas) were also noted.

A consistent concern was that agrovet shops stock veterinary drugs alongside general merchandise, and that some pharmacies and even farmers' own shops are entering the veterinary drug supply chain without adequate oversight:

*"Even the farmers' shops, they also stock these veterinary drugs."* — Ndola Senior VO

*"ADS chemist... Kafubu Agrovet... Luanshya agro shop — so the Copperbelt Province at large, they are veterinary agro shops."* — Ndola Senior VO

The profit motive of agrovets was highlighted as a systemic driver of inappropriate antibiotic dispensing without prescription:

*"They want to sell as much and make their target. So they will just sell the farmer whatever the farmer needs."* — Ndola Senior VO

*"Usually they want to offload products and won't bother to get prescriptions or ask for them. They want to sell these drugs within the shortest possible time — in short they want to make profit."* — Chongwe District Coordinator

## 3.2 Farmer Antimicrobial Use Practices

All three informants described a consistent pattern of self-treatment by farmers, driven by previous experience, neighbour advice, and agrovet-guided selection, rather than by professional prescription. Key concerns raised include:

### Non-adherence to withdrawal periods

Withdrawal periods were acknowledged as poorly understood and inconsistently observed. The Ndola DVO reported that farmers were 'shocked' to learn about withdrawal periods during post-mortem consultations, suggesting this is a foundational knowledge gap:

*"When you tell them that they shouldn't give drugs, they shouldn't sell animals immediately after — most of them will be shocked. It's like they've never heard that there's a withdrawal period and what it means and all that."* — Ndola DVO

*"Farmers do follow withdrawal periods, but I think withdrawal periods are kind of tricky... they are usually under pressure to offload their stock onto the Market."* — Chongwe District Coordinator

### Absence of prescriptions

Prescription use is not standard practice. Farmers typically bypass veterinary consultation entirely, going directly to agrovets based on neighbour recommendation:

*"Their neighbour said, I gave this and it's working. And that is how withdrawal periods are not followed unless you emphasise it."* — Ndola DVO

*"For prescriptions also, it's not — because of the business nature of the supply chain."* — Chongwe District Coordinator

### Incorrect dosing

Underdosing to save money was reported in Chongwe, and both overdosing and underdosing are observed on the Copperbelt. Prophylactic use — administering antibiotics before any signs of illness — was also documented:

*"Due to pressure, farmers at times underdose to save money."* — Chongwe District Coordinator

*"I had a case of a farmer who came to say, my pigs died last time — so I've decided to buy this particular drug and just administer before they get sick."* — Ndola Senior VO

### Irrational antibiotic switching

Treatment failure is often managed by changing drugs based on neighbour advice or agrovet suggestion rather than diagnostic confirmation. Farmers expect rapid results and change antibiotics within 24–48 hours if no improvement is seen:

*"If they don't get better in two days, they will change the drug. So because the neighbour said this... they don't even say what they were treating for."* — Ndola DVO

## 3.3 Storage Practices for Antimicrobials and Vaccines

Storage quality varies significantly by actor type. Key findings:

- Agrovet shops: Generally have adequate shade and room-temperature storage for solid antimicrobials, but cold chain management for vaccines is inadequate in many outlets.
- Government veterinary offices: Reported to have acceptable storage facilities for office use.
- Farmers: Store antibiotics on shelves in their homes or animal sheds without temperature control. Vaccine storage is particularly problematic due to cold chain requirements.
- Para-veterinarians and lab assistants: Described as keeping drugs 'anyhow' with little regard for storage protocols, prioritising income over quality.

*"For the vet assistants and lab assistants, they just keep them anyhow because they are looking at the money they will make."* — Ndola Senior VO

*"There is a concern that farmers are told to combine Newcastle and Gumboro vaccine at the same time — so really they are not adhering, maybe due to lack of knowledge from the agro shop because the agro shops themselves are not employing the right personnel."* — Ndola Senior VO

A structural gap was noted: the Chongwe coordinator suggested that solar-powered communal cold storage could be a practical solution for rural areas:

*"In some areas, especially when it comes to governments, we can provide provision of solar in communal places where they can store these vaccines. It can actually be helpful."* — Chongwe District Coordinator

## 3.4 Expiry Date Compliance

Expiry date compliance is uneven and enforcement is minimal. Commercial farmers were noted to be more vigilant, while small-scale and rural farmers rarely check labels:

*"Some of our farmers — because of the lack of knowledge — won't bother to check. Very few do go into detail and check the expiry dates. Enforcement is needed. We also need to go into these shops to ensure all the dates for the drugs are within expiry."* — Chongwe District Coordinator

*"For small-scale farmers, maybe they are far from the agro shop and they won't even read to say, okay, let me read on the bottle label that this drug has expired."* — Ndola Senior VO

ZEMA (Zambia Environmental Management Agency) was positively noted for providing training to agrovets on disposal of expired medicines, and this appeared to be gaining some traction among Copperbelt agrovets. However, the reach of such enforcement is uneven across districts.

## 3.5 Record Keeping

Record keeping among poultry farmers is limited and incomplete. Where records are kept, they typically cover vaccination schedules and flock acquisition. Treatment records — including the names of drugs used, the indication (disease being treated), dosage, and duration — are rarely maintained:

*"They wouldn't tell you what treatment they give. They'll say 'this medicine' but won't say what they were treating for. They were just giving so that the chickens don't continue dying."* — Ndola DVO

*"Whatever they buy they will keep records of the stock number. They also age the chickens because they're interested in vaccinations — but few keep records of treatments, deaths, and the actual drugs used."* — Chongwe District Coordinator

All informants agreed that targeted training on record keeping is needed, particularly for small-scale and backyard farmers.

## 3.6 Treatment Failure and AMR Experiences

All three informants acknowledged encountering or hearing reports of treatment failure, though their direct experiences varied:

- Chongwe District Coordinator: Reported common experiences of treatment failure, particularly in cases of mastitis where farmers cycle through multiple antibiotics without diagnostic guidance.
- Ndola DVO: Described treatment failure as an escalating phenomenon where farmers change drugs rapidly without professional consultation, rather than seeking a diagnosis.
- Ndola Senior VO: Described a personal and memorable experience of AMR in human medicine — her child's tonsillitis requiring sequential escalation from amoxicillin to amoxicillin-clavulanate before responding — which grounded her AMR understanding in concrete terms.

*"I've seen it and I've experienced it on my children... They kept on prescribing one antibiotic after the other. No response. Yeah."* — Ndola Senior VO

*"In cases of mastitis, farmers use all types of antibiotics, then you ask them why they've been changing antibiotics and they can't give you reasons."* — Chongwe District Coordinator

The professional response to treatment failure varied by informant. The Chongwe coordinator reported advising diagnostic sampling (culture and sensitivity testing), while the Ndola Senior VO described an integrated approach combining post-mortem, management review, and drug review. The Ndola DVO reflected on the systemic problem that treatment failure is only reported after significant mortality, by which time the diagnostic window has passed.

## 3.7 AMR Knowledge, Awareness, and Training Gaps

### Among veterinary professionals

All informants confirmed familiarity with the concept of AMR, though the depth of understanding varied considerably. Common sources of AMR knowledge included: university training (for some), workshops, and self-directed learning through journals, internet, and experience. Notably, the longest-serving respondent (Ndola DVO) stated she could not remember AMR being taught at university 25+ years ago:

*"At school I don't know... I don't remember hearing anything on antimicrobial resistance."* — Ndola DVO

Professionals rated their own community's understanding of AMR between 40% and 60%, with two informants emphasising a critical gap between stated knowledge and practical behaviour:

*"Among the professionals, we might think that we know more — but in reality the understanding of AMR is more than we know... there are times when we claim as professionals that we know more, but in practice we are the ones who at times perpetuate the emergence or the transmission."* — Chongwe District Coordinator

*"I'm yet to see them practising that and advising lesser use of these antimicrobials."* — Ndola DVO

### Among farmers

AMR awareness among farmers was estimated at extremely low levels — ranging from 10% to 45% — across the three respondents. The Ndola Senior VO's estimate of 10% reflects her observations that farmers continue to demand antibiotics even for non-infectious presentations, and that training messages are not being retained or disseminated:

*"I had someone come here with a chicken that wasn't sick to me — but they wanted an antibiotic. I had to convince them that they actually don't need to use an antibiotic."* — Ndola DVO

*"I don't know if they are able to disseminate the information to their fellow farmers... because I'm still seeing the same practice."* — Ndola DVO

### Training gaps

A consistent finding was that university-level training on AMR is insufficient and largely delivered within the context of microbiology (typically year 3), without adequate emphasis on practical AMR stewardship:

*"Remember, we are talking more microbiology, which is more in the third year. But they could add more to the syllabus."* — Chongwe District Coordinator

*"For paraprofessionals, it is also there but done on the surface, so no wonder they'll need more CPDs."* — Chongwe District Coordinator

A particularly insightful observation from the Ndola DVO concerned the framing of AMR training: presenting AMR as a 'panic' situation caused disengagement rather than engagement among participants:

*"When you're presenting the message to people, present it in a way that... when you present in a certain way, they get scared. And when some people react differently when they get scared, so they would say they don't want to — they probably don't even want to know about it because they're scared."* — Ndola DVO

## 3.8 Treatment Guidelines

None of the three informants had access to finalised veterinary treatment guidelines at the time of interview. All were aware of efforts to develop guidelines. The Ndola Senior VO reported that the Ministry of Livestock (Department of Field Services, through Dr Goma) had indicated that guidelines were 'almost done' and would be disseminated to provinces and districts upon completion. The Ndola DVO reported searching for guidelines after attending a Lusaka workshop and finding only human health guidelines — none for animals:

*"After that I checked, I couldn't find any on animals, but I found a lot on humans."* — Ndola DVO

*"I've heard about it — for poultry, beef, small animals, and more interestingly, aquaculture."* — Chongwe District Coordinator

The absence of accessible, disseminated treatment guidelines for animal species — particularly poultry — is a critical gap that directly enables irrational antibiotic use among professionals and agrovets alike.

# 4. Cross-Cutting Synthesis

The thematic findings converge around a set of interconnected system-level failures that cannot be addressed in isolation. The table below summarises the major themes, key findings, illustrative evidence, and priority recommendations.

| **Theme** | **Key Finding** | **Evidence from Transcripts** | **Recommendation** |
| --- | --- | --- | --- |
| **Drug supply & dispensing** | Agrovets are the dominant point of AMU, operating largely without prescription requirements due to commercial incentives. | 'They want to sell as much and make their target.' (Ndola Senior VO) | Strengthen ZEMA/MoL inspection; enforce prescription-only dispensing; register all veterinary drug outlets. |
| **Farmer AMU practices** | Self-treatment, dosing errors, antibiotic prophylaxis, and non-adherence to withdrawal periods are widespread. | 'Farmers are under pressure to offload... at times they won't come out clean to the consumers.' (Chongwe DC) | Community-level AMU education; targeted withdrawal period campaigns; farmer-friendly AMR messaging. |
| **Storage & cold chain** | Vaccine cold chain is inadequate especially for rural farmers; para-professionals store drugs inappropriately. | 'They just keep them anyhow because they are looking at the money.' (Ndola Senior VO) | Invest in communal solar cold storage; strengthen agrovet storage audits; mandatory training for para-professionals. |
| **Expiry date compliance** | Small-scale farmers rarely check expiry dates; enforcement of agrovets is improving but geographically patchy. | 'Enforcement is needed. We need to go into these shops to ensure all the dates are within expiry.' (Chongwe DC) | Extend ZEMA/ZAMRA inspection to all districts; simple label literacy campaigns for farmers. |
| **Record keeping** | Treatment records are absent; vaccination and acquisition records are kept but incomplete. | 'They won't say what they were treating for. They were just giving so that the chickens don't continue dying.' (Ndola DVO) | Introduce simple poultry health record cards; integrate record keeping into farmer training curricula. |
| **Treatment failure & AMR experience** | Treatment failure is common but underreported and addressed without diagnostics; AMR is real but poorly quantified. | 'We only get to hear about it long after a lot of animals are sick or dead.' (Ndola DVO) | Establish sentinel surveillance for treatment failure; promote culture & sensitivity testing; link clinical observations to AMR data. |
| **AMR knowledge & training** | AMR awareness among professionals is 40–60% and among farmers as low as 10%; university training is insufficient; CPD is critical. | 'We are the ones who at times perpetuate the emergence.' (Chongwe DC) | Mandatory AMR CPD for all licensed animal health professionals; curriculum review at UNZA & animal health colleges; reframe AMR messaging to avoid panic. |
| **Treatment guidelines** | No finalised, accessible veterinary treatment guidelines exist; professionals are aware of development but do not have access. | 'I couldn't find any on animals, but I found a lot on humans.' (Ndola DVO) | Urgently finalise and disseminate MoL treatment guidelines to all provinces and districts; promote their use in agrovet training. |

# 5. Prioritised Recommendations

Priority 1 — Immediate actions (0–3 months)

- Finalise and distribute the MoL veterinary treatment guidelines to all provinces, districts, and registered agrovets as a matter of urgency — this is the single most-requested and most impactful systemic intervention.
- Launch a targeted withdrawal period awareness campaign for broiler farmers, co-facilitated by DVOs, linked to market entry points and slaughter facilities.
- Convene a joint ZAMRA / MoL inspection blitz of agrovet shops in Chongwe and Ndola to assess prescription practices, storage conditions, and expiry date compliance.

Priority 2 — Short-term actions (3–6 months)

- Design and pilot a Continuing Professional Development (CPD) module on AMR stewardship for all registered veterinary officers and veterinary assistants in both provinces. CPD should include behaviour change components, not just knowledge transfer.
- Introduce a simple, one-page poultry health record card (flock size, date of entry, vaccination dates, treatments given with drug name and indication, withdrawal date, mortality) and integrate into existing farmer training visits.
- Review and update AMR content in the University of Zambia veterinary curriculum and Zambia Animal Health College training programmes, in partnership with the MoL and WHO/FAO.

Priority 3 — Medium-term actions (6–18 months)

- Invest in solar-powered communal cold storage facilities in rural Chongwe and Copperbelt communities to address the critical vaccine cold chain gap identified by all three informants.
- Establish a treatment failure sentinel surveillance system, building on existing post-mortem services at district veterinary offices. Link clinical data to a national AMR data repository.
- Develop and deploy an AMR communication strategy that reframes the issue from a 'panic' narrative to a 'solvable problem with local actions' — drawing on the communications critique raised by the Ndola DVO.
- Engage ZAMRA to scale up veterinary drug registration and inspection nationally, with particular attention to informal drug distribution channels identified in this study.
- Explore antibiotic-free or reduced-antibiotic poultry production models as a positive alternative to incentivise, rather than relying solely on regulatory restrictions.

# 6. Conclusions

These Key Informant Interviews reveal a system under significant AMR risk, driven by interconnected failures in regulation, education, and professional practice. The picture that emerges is consistent across two provinces and three different veterinary roles: antimicrobials are widely and easily available without prescription through a commercial agrovet network; farmers self-treat based on prior experience and neighbour advice; professionals understand AMR conceptually but often perpetuate the same irrational practices they observe in farmers; and the national infrastructure for AMR surveillance, treatment guidelines, and cold chain logistics is under-developed.

What distinguishes this study from purely quantitative surveys is the texture of insight offered by the informants. The Ndola Senior VO's personal encounter with AMR in her child's care, the Chongwe coordinator's candid admission that professionals 'perpetuate the emergence', and the Ndola DVO's observation that panic-framed AMR messaging causes disengagement — these are actionable insights that quantitative data alone would not surface.

The good news, articulated by all three informants, is that the problem is understood and the solutions are known: accessible treatment guidelines, targeted farmer education, enforced prescription policies, and sustained CPD for professionals. The CAAZAP project is well-positioned to translate these insights into concrete interventions that can reduce AMR risk across both provinces.

Key quote: "The problem is real. And it's there both in humans and animals. I think it's a collective effort from the animal health sector, the human health sector, even the environmental experts." — Ndola Senior Veterinary Officer, Copperbelt Province

# Appendix: Summary of Interview Details

| **Detail** | **Interview 1** | **Interview 2** | **Interview 3** |
| --- | --- | --- | --- |
| **Label** | KII — Chongwe District Coordinator | Ndola DVO | Ndola Senior Vet (Copperbelt SVO) |
| **Name** | Dr Francis Mwanza | Anonymous | Dr Caroline |
| **Title / Role** | District Coordinator, Chongwe | District Veterinary Officer, Ndola | Senior Veterinary Officer, Copperbelt Province |
| **Location** | Chongwe District, Lusaka Province | Ndola, Copperbelt Province | Ndola, Copperbelt Province |
| **Interviewer** | Chikwanda Chileshe | Chikwanda Chileshe | Chikwanda Chileshe |
| **Project** | CAAZAP | CAAZAP | CAAZAP |
| **Registration** | VAZ and VCZ | VAZ/VCZ (confirmed) | VAZ (mandatory via PMD) |
| **AMR knowledge est. – vets** | ~60% | ~51% | ~40% |
| **AMR knowledge est. – farmers** | <50% | ~45% | ~10% |
| **Key concern raised** | Profit-driven agrovets; underdosing; enforcement gaps | Withdrawal period ignorance; irrational drug switching | Para-professional misconduct; cold chain failures; behaviour change needed |

*Note: All interview content is reported as faithfully as possible from available transcripts. Some portions of transcripts contained speech recognition artefacts or unclear audio segments (noted as '...' in original transcripts). Quotes have been lightly edited for clarity while preserving meaning. Anonymity has been preserved where informants did not provide names.*
