# Supplementary material for: Bridging knowledge and practice gaps in antimicrobial stewardship among veterinary professionals in Zambia’s poultry sector: a mixed-methods pre–post intervention study
Source: Front Antibiot. 2026 Jul 30;5:1898867. doi: 10.3389/frabi.2026.1898867 (PMC13469284; doi:10.3389/frabi.2026.1898867)
Supplement: Supplementary file 3 [file Table3.docx]

Consolidated Criteria for Reporting Qualitative Research (COREQ) checklist for the key informant interview component.

|  | **COREQ item** | **How they are addressed** |
| --- | --- | --- |
| **Domain 1 — Research team and reflexivity** | | |
| 1 | Interviewer / facilitator | All three KIIs were conducted by a single interviewer (C.C.). |
| 2 | Credentials | Master of Science (MSc) in Public Health. |
| 3 | Occupation | Public health researcher and member of the CAZAAP project research team at the time of the study. |
| 4 | Gender | Female. |
| 5 | Experience and training | Completed training in qualitative interviewing techniques prior to data collection; experienced in CAZAAP project fieldwork. |
| 6 | Relationship established | No close personal relationship preceded the interviews; interviewer and informants were linked only through the CAZAAP project. |
| 7 | Participant knowledge of the interviewer | Informants were told the interviewer was collecting data as part of the CAZAAP project’s evaluation of antimicrobial stewardship. |
| 8 | Interviewer characteristics (assumptions, interests) | Interest in antimicrobial stewardship as a project team member; addressed through the reflexivity statement (open, non-leading questioning; analytic decisions discussed within the team). |
| **Domain 2 — Study design** | | |
| 9 | Methodological orientation and theory | Qualitative description with thematic analysis, embedded in a convergent mixed-methods design. |
| 10 | Sampling | Purposive sampling of senior veterinary professionals with relevant experience and familiarity with the districts served. |
| 11 | Method of approach | Informants approached in person through the CAZAAP project network. |
| 12 | Sample size | Three key informants. |
| 13 | Non-participation | No approached informant declined or withdrew. |
| 14 | Setting of data collection | Informants’ duty stations in Chongwe District (Lusaka Province) and Ndola (Copperbelt Province). |
| 15 | Presence of non-participants | Interviews conducted one-to-one; no other persons present. |
| 16 | Description of sample | A District Coordinator/veterinarian (Chongwe); a District Veterinary Officer (Ndola); and a Senior Veterinary Officer for Copperbelt Province (Ndola). Profiles reported in Methods and Results. |
| 17 | Interview guide | Semi-structured interview guide developed by the research team. Piloting was done in Kabwe. |
| 18 | Repeat interviews | No repeat interviews were conducted. |
| 19 | Audio / visual recording | Audio-recorded using digital voice recorders and mobile phones, with separate consent for recording. |
| 20 | Field notes | No Field notes were taken |
| 21 | Duration | Approximately 20 to 30 minutes per interview. |
| 22 | Data saturation | Interviews continued until no substantially new major themes emerged; as only three senior informants were available in-district, full saturation is not claimed and the KIIs are presented as a complementary explanatory strand. |
| 23 | Transcripts returned | Transcripts were not returned to participants for comment. |
| **Domain 3 — Analysis and findings** | | |
| 24 | Number of data coders | 1. |
| 25 | Description of the coding tree | Broad a priori thematic areas (knowledge, attitudes, practices) were elaborated into specific and emergent sub-themes; coding managed in MAXQDA. |
| 26 | Derivation of themes | Both predefined (KAP domains) and inductively derived from the data. |
| 27 | Software | MAXQDA. |
| 28 | Participant checking | Participants did not provide feedback on the findings. |
| 29 | Quotations presented | Representative verbatim quotations, identified by informant role, are now presented for each theme. |
| 30 | Data and findings consistent | Findings are substantiated by quoted data and triangulated with quantitative results in the joint display (Table 5). |
| 31 | Clarity of major themes | Major themes are presented under clear headings in the Results. |
| 32 | Clarity of minor themes | Minor / divergent themes (e.g. “panic”-framed messaging causing disengagement; cold-chain and storage failures) are described. |
